# A Context-Dependent Role for MiR-124-3p on Cell Phenotype, Viability and Chemosensitivity in Neuroblastoma *in vitro*

**DOI:** 10.3389/fcell.2020.559553

**Published:** 2020-11-20

**Authors:** John C. Nolan, Manuela Salvucci, Steven Carberry, Ana Barat, Miguel F. Segura, Justine Fenn, Jochen H. M. Prehn, Raymond L. Stallings, Olga Piskareva

**Affiliations:** ^1^School of Pharmacy and Biomolecular Sciences, RCSI University of Medicine and Health Sciences, Dublin, Ireland; ^2^National Children’s Research Centre, Our Lady’s Children’s Hospital Crumlin, Dublin, Ireland; ^3^Department of Physiology and Medical Physics and RCSI Centre for Systems Medicine, RCSI University of Medicine and Health Sciences, Dublin, Ireland; ^4^Group of Translational Research in Child and Adolescent Cancer, Vall d’Hebron Research Institute, Barcelona, Spain; ^5^Department of Anatomy and Regenerative Medicine, RCSI University of Medicine and Health Sciences, Dublin, Ireland

**Keywords:** miRNA – microRNA, miR-124-3p, neuroblastoma, drug-resistance, cytoskeleton, cellular phenotype plasticity

## Abstract

Neuroblastoma (NB) is a neural crest-derived tumor, which develops before birth or in early childhood, with metastatic dissemination typically preceding diagnosis. Tumors are characterized by a highly heterogeneous combination of cellular phenotypes demonstrating varying degrees of differentiation along different lineage pathways, and possessing distinct super-enhancers and core regulatory circuits, thereby leading to highly varied malignant potential and divergent clinical outcomes. Cytoskeletal reorganization is fundamental to cellular transformations, including the processes of cellular differentiation and epithelial to mesenchymal transition (EMT), previously reported by our lab and others to coincide with chemotherapy resistance and enhanced metastatic ability of tumor cells. This study set out to investigate the ability of the neuronal miR-124-3p to reverse the cellular transformation associated with drug resistance development and assess the anti-oncogenic role of this miRNA in *in vitro* models of drug-resistant adrenergic (ADRN) and mesenchymal (MES) neuroblastoma cell lines. Low expression of miR-124-3p in a cohort of neuroblastomas was significantly associated with poor overall and progression-free patient survival. Over-expression of miR-124-3p *in vitro* inhibited cell viability through the promotion of cell cycle arrest and induction of apoptosis in addition to sensitizing drug-resistant cells to chemotherapeutics in a panel of morphologically distinct neuroblastoma cell lines. Finally, we describe miR-124-3p direct targeting and repression of key up-regulated cytoskeletal genes including *MYH9*, *ACTN4* and *PLEC* and the reversal of the resistance-associated EMT and enhanced invasive capacity previously reported in our *in vitro* model (SK-N-ASCis24).

## Introduction

Neuroblastoma is pediatric cancer occurring predominantly in children under 5 years with highly variable clinical outcomes. Genomic aberrations, such as *MYCN* amplification, *ALK/NRAS* mutation or p53, chromosome 11q loss, patient age at diagnosis, disease stage, and degree of tumor differentiation are all predictive of patient outcome (Davidoff, 2012; Louis and Shohet, 2015). Despite improvements in patient survival with recently developed immunotherapies, a substantial proportion of neuroblastoma patients either do not respond to treatment or relapse with the acquisition of drug resistance and an overall survival probability of 20% (Yu et al., 2010; Gatta et al., 2014; Berlanga et al., 2017; Erbe et al., 2018). Therefore, a better understanding of the mechanisms underlying neuroblastoma progression is needed.

Neuroblastoma displays significant intra-tumor cellular heterogeneity which governs response to treatment and the cellular landscape comprising the tumor, making long term success in the treatment of this aggressive pediatric disease particularly challenging. Understanding the different cell populations which exist within tumors and how their cytoskeletal and morphological interconversion correlates with drug response, metastatic potential and disease progression offers valuable insight for the advance of neuroblastoma research.

MiRNA are well established to act as post-transcriptional regulators of genes involved in a diverse array of biological processes including cell proliferation, differentiation (mir-10a/b, let-7a), cell cycle progression, apoptosis (miR-34a, mir-184), and chemo-resistance (miR-497, miR-204) (Foley et al., 2010, 2011; Tivnan et al., 2010, 2011, 2012; Lynch et al., 2012, 2013; Molenaar et al., 2012; Ryan et al., 2012; Creevey et al., 2013; Domingo-Fernandez et al., 2013). Clinically, aberrant miRNA expression is associated with tumorigenesis with abundant studies demonstrating miRNA acting as tumor suppressors or oncomiRs (Bray et al., 2009; Mestdagh et al., 2010; Schulte et al., 2010; De Preter et al., 2011). MiRNA also acts as determinants of cell morphology, with specific neuronal (miR-124, miR-375) and non-neuronal (miR-21, miR-221, and miR-335) miRNA abundantly expressed in panels of morphologically distinct neuroblastoma cell lines (Samaraweera et al., 2014). Consequently, this study set out to identify and assess a miRNA involved in disease progression and cellular transformation.

The cytoskeletal genes *MYH9*, *PLEC*, *ITGB1*, *VIM*, and *ACTN4*, were previously reported by our lab to drive EMT-like morphological transformation and increased invasiveness (2.5 fold) in the MES SK-N-ASCis24 neuroblastoma cell line, coinciding with resistance development (Piskareva et al., 2015). This study reported a 5.3 fold increase in cisplatin resistance with cross-resistance to the topoisomerase inhibitors; etoposide (2.3 fold) and irinotecan (5.4 fold) in the SK-N-ASCis24 cell line. The panel of cytoskeletal genes was identified as targets of the neuronal miR-124-3p by combined computational prediction software leading us to postulate that miR-124-3p could reverse or inhibit the reported morphological transformation.

In this study, we report a significant association of low miR-124-3p expression with poor overall and progression-free survival in a cohort of neuroblastoma tumors of various stage and clinical factors. We demonstrate direct targeting and knockdown of our panel of upregulated cytoskeletal genes by miR-124-3p resulting in a reversal of resistance-associated EMT and invasion. We also demonstrate the potential of miR-124-3p to inhibit proliferation and viability in a panel of neuroblastoma cell lines of different morphological subtype *in vitro*. Finally, we show mild levels of cell cycle arrest, apoptosis induction and resensitization to chemotherapy in miR-124-3p transfected neuroblastoma cells, supporting an overall cancer suppressive role in neuroblastoma. Collectively, this data supports a significant context dependant anti-cancer role for miR-124-3p in neuroblastoma cell lines *in vitro*.

## Results

### Association of Low MiR-124-3p Expression With Reduced Patient Survival

**FIGURE 1:**
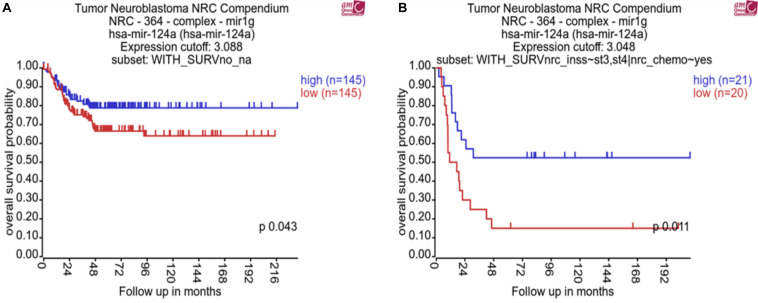
Kaplan–Meier plots showing miR-124-3p clinical relevance on overall survival in **(A)** a cohort of patients with neuroblastoma tumors of varying stage before chemotherapy (*n* = 290) and **(B)** a cohort of patients with high risk neuroblastoma tumors following chemotherapy (*n* = 41). *P*-values were obtained using log-rank test.

In order to assess the clinical relevance of miR-124-3p in neuroblastoma, we analyzed whether expression was associated with patient survival, international neuroblastoma staging system (INSS), and common chromosomal abnormalities. This analysis was conducted using the neuroblastoma research consortium (NRC) tumor cohort with the R2 data analysis platform (AMC). Kaplan–Meier analysis of chemotherapy-naive neuroblastoma tumors of varying stages (*n* = 290) and post-chemotherapy high-risk neuroblastoma tumors (*n* = 41) from this cohort identified a significant association of low miR-124-3p expression with poor overall survival (Figure 1).

MiRNA-124-3p expression was significantly lower in high risk stage 4 (*n* = 122) tumors compared to tumors of lower stages (1, 2, and 3, *n* = 139) (*p* = 0.0035). Expression of miR-124-3p was also found to be significantly lower in *MYCN* diploid stage 4 tumors (*n* = 79) relative to *MYCN* amplified tumors of the same stage (*n* = 43; *p* = 0.0002) (Supplementary Material S1). We evaluated the association of miR-124-3p expression with clinical outcome via Univariate Cox proportional hazards regression. Three additional risk factors: *MYCN* amplification (yes, no), INSS (Stage 1, 2, 3, 4, 4S) and 11q deletion (yes, no) were also assessed for association with clinical outcome via univariate Cox proportional hazards regression. Univariate analyses with respect to *MYCN* amplification, stage and 11q deletion reported statistically significant associations with clinical outcome (Supplementary Material S2A). MiR-124-3p was not significantly associated with survival time [in both continuous and discreet (high, >median vs. low, <median) univariate Cox regression]. Multivariate Cox regression was run with the covariates *MYCN* amplification, stage and deletion of 11q (Supplementary Material S2B). This analysis found that 11q deletion was not significantly associated with survival time when adjusting for *MYCN* amplification and stage. Because of the limited number of patients with known status of 11q deletion and confounding between stage and 11q deletion, we excluded this risk factor from further analysis and carried out multivariate Cox regression encompassing 290 patients and including the following covariates: *MYCN* amplification and stage at diagnosis (grouping lower stages), Also, because of assumption of proportional hazards not being respected for the stage, we stratify the model based on this variable. The hazard ratios obtained for *MYCN* amplification for the stage stratified model is shown in Supplementary Material S2C. These results, while demonstrating miR-124-3p does not act independently as a prognostic indicator for neuroblastoma, it nonetheless has an evident involvement in disease progression and outcome.

### MiR-124-3p Reduces Neuroblastoma Cell Viability *in vitro*

**FIGURE 2:**
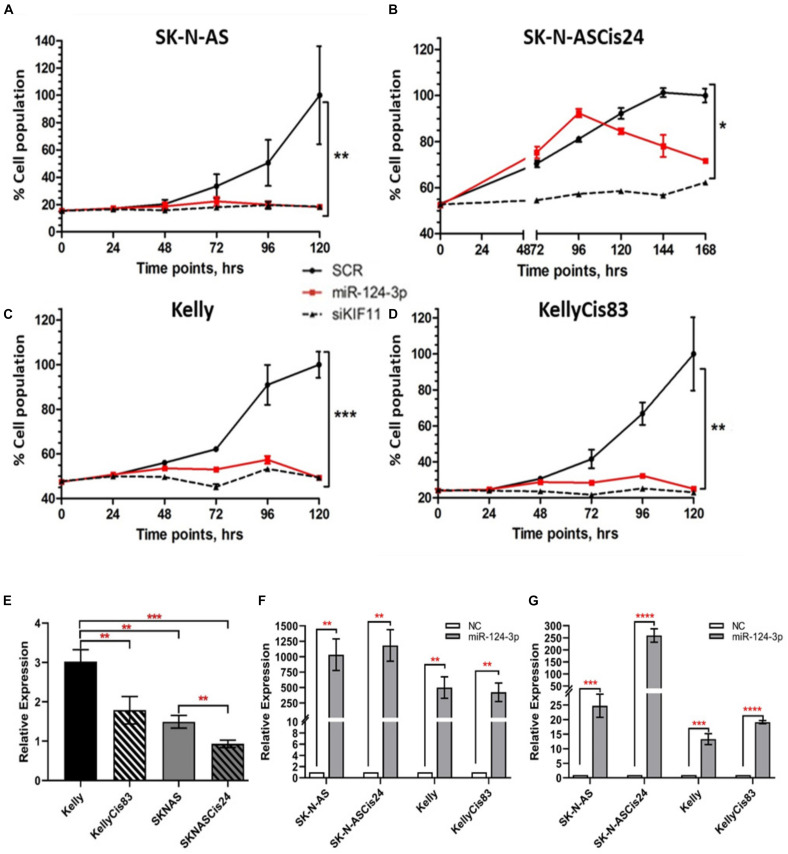
**(A–D)** Cell viability was determined by acid phosphatase assay at 24 h, 48 h, 72 h, 96 h, and 120 h post transfection for the SK-N-AS/Kelly/KellyCis83 cell lines and up to 168 h for the SK-N-ASCis24 cell line. **(E)** Relative endogenous expression of miR-124-3p in neuroblastoma cell lines. **(F,G)** Expression of miR-124-3p in miRNA transfected neuroblastoma cell lines at 48 h and 96 h, respectively, normalized to negative control transfected cells. Asterisks indicate statistical significance obtained using unpaired Student’s *t*-test (*p < 0.05, **p < 0.01, ***p < 0.001, ****p < 0.0001).

In view of the clinical relevance of miR-124-3p in neuroblastoma, we assessed cell growth and viability in response to over-expression. Transfection with miR-124-3p mimics resulted in a significant reduction in relative cell number and viability across all neuroblastoma cell lines tested. An increase in doubling time was previously reported by our lab in the SK-N-ASCis24 cell line following cisplatin resistance development and as a result cell growth and viability was assessed at a later time point than that of the corresponding parental SK-N-AS parental cell line (Piskareva et al., 2015). By assessing cell viability every 24 h following transfection in each cell line up to 120 h and 168 h for SK-N-ASCis24, we demonstrated suppression of cell viability as early as 48 h, up until the final time point. Of the cell lines tested SK-N-AS (*p* = 0.0058), KellyCis83 (*p* = 0.0078), and Kelly (*p* = 0.0004) demonstrated the most substantial growth inhibitory effect in response to miR-124-3p expression, showing levels equal to the siKiff11 positive control. Inhibition of cell viability in the SK-N-ASCis24 (*p* = 0.0277) did not occur until approximately 96 h post-transfection, increasing until the final time point; however, never demonstrating the same degree of suppression of cell viability as that observed in any other cell lines tested and only causing 80% of the inhibition seen in this cell line in response to a positive control (Figures 2A–D). Endogenous expression of miR-124-3p was determined for each cell line demonstrating lowest levels in the SK-N-ASCis24 cell line with 3-fold higher expression in the Kelly cell line, representing the highest expression of the cell lines tested (Figure 2E). Relative expression following transfection with miR-124-3p was assessed at 48 h and 96 h, demonstrating a 500–1000 fold and 25–250 fold increase, respectively, compared to cells transfected with scrambled oligonucleotides (Figures 2F,G). These results informed our selection of the optimal time points for analysis of cell cycle distribution and apoptosis induction across our different neuroblastoma cell lines in response to miR-124-3p expression *in vitro*.

### Overexpression of MiR-124-3p Targets Key Morphologically Determinant Genes and Alters Neuroblastoma Cell Morphology *in vitro*

**FIGURE 3:**
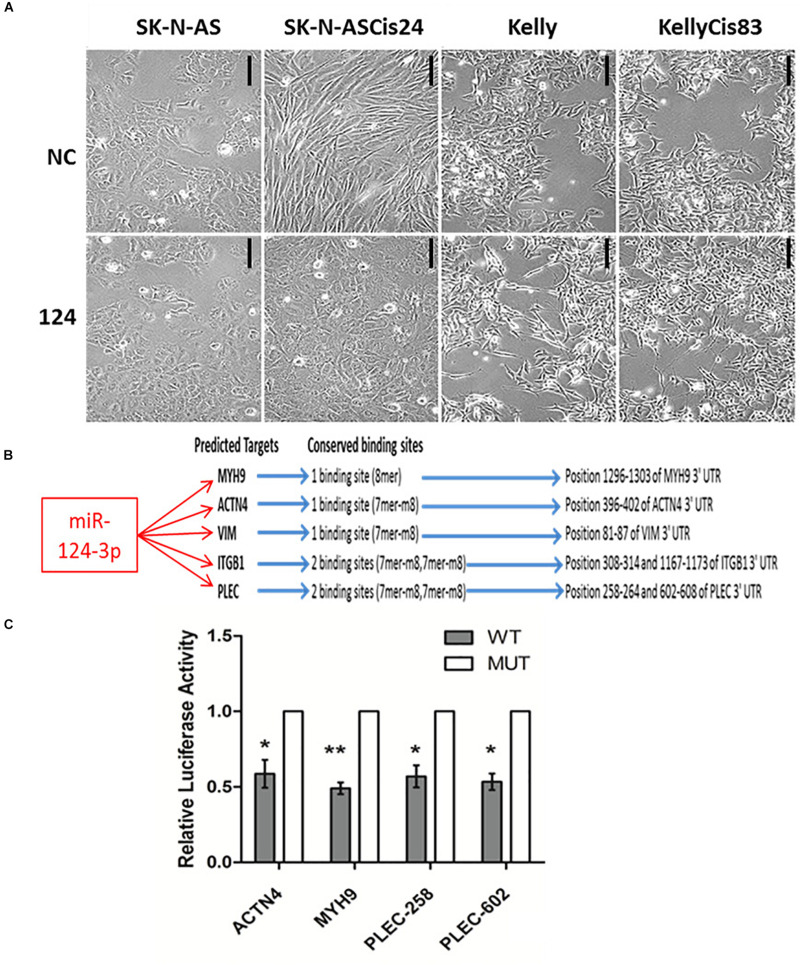
**(A)** Photomicrographs of SK-N-AS, SK-N-ASCis24, Kelly, and KellyCis83 neuroblastoma cells transfected with miR-124-3p or scrambled control (NC) for 48 h (Scale bar 50 μm). **(B)** Conserved miR-124-3p target binding sites. **(C)** Luciferase activity determined in Kelly neuroblastoma cells at 48 h after co-transfection with negative control or miR-124-3p mimic and either wild-type (WT) or mutated (MUT) ACTN4, MYH9, and PLEC 3′UTR constructs. Asterisks indicate statistical significance obtained using unpaired Student’s *t*-test (*p < 0.05, **p < 0.01, ***p < 0.001, ****p < 0.0001).

Neuroblastoma consists of adrenergic and mesenchymal cancer cell types, which have distinct phenotypes, super-enhancers and core regulatory circuitries which drive the substantial clinical heterogeneity associated with this disease (Ciccarone et al., 1989; van Groningen et al., 2017; Szemes et al., 2019). In order to investigate the functional role of miR-124-3p in morphologically and phenotypically distinct neuroblastoma cell lines, we examined the effect of miR-124-3p over-expression on the adrenergic Kelly/KellyCis83 and mesenchymal SK-N-AS/SK-N-ASCis24 neuroblastoma cell lines. Transfection with miR-124-3p resulted in an enhanced epithelial-like phenotype in the parental SK-N-AS neuroblastoma cell line when expressed at hyper-physiological levels. The cisplatin-resistant SK-N-ASCis24 cell line, however, demonstrated a substantial cytoskeleton defined morphological reversion from an elongated mesenchymal morphology to the more flattened polygonal epithelial morphology of its parental SK-N-AS cell line before resistance-associated EMT-like cellular transformation. Interestingly, miR-124-3p expression provoked a different response in the adrenergic lineage committed Kelly and KellyCis83 cell lines, with cells growing in aggregates and exhibiting lower substrate adherence and increased neurite-like outgrowths from the cell body, indicating an enhanced adrenergic cell phenotype (Figure 3A). This difference in morphological response between cell lines is likely due to the highly varied epigenetic landscape and expression of cytoskeletal genes, many of which are targeted by miR-124-3p either directly or via network partners and which are regulated by competing gene regulatory networks between cell types (Boeva et al., 2017; Szemes et al., 2019; Veschi et al., 2019; Upton et al., 2020).

Expression of a panel of cytoskeletal proteins including the non-muscle myosin II (MYH9) was previously reported by our lab to be significantly up-regulated in the drug-resistant mesenchymal neuroblastoma cell line, SK-N-ASCis24, and shown to be central to ROCK1-independent cytoskeletal restructuring and increased migratory potential following cisplatin selection (Piskareva et al., 2015). In order to validate the ability of miR-124-3p to modulate this cellular transformation associated with cisplatin resistance in SK-N-ASCis24, we performed a 3′ UTR analysis for potential binding sites on *MYH9* mRNA. Cross-examining predictions between TargetScan, Miranda (score > 60) and PITA (ddG cutoff −5) identified miR-124-3p binding sites in the 3′ UTR of *MYH9* mRNA by all three algorithms (Kertesz et al., 2007). In addition to its predicted targeting of *MYH9*, miR-124-3p was predicted to target additional cytoskeletal genes (*PLEC*, *ITGB1*, *VIM*, and *ACTN4*) from our panel, which were overexpressed in the resistant SK-N-ASCis24 neuroblastoma cell model. These cytoskeletal genes were not independently significantly associated with overall survival in neuroblastoma; however, they were of particular interest because of their demonstrated involvement in cytoskeletal remodeling, cell-cell junctions, adhesion and migration as previously reported by Ingenuity Pathway Analysis (Piskareva et al., 2015). MiR-124-3p has one conserved 8-mer seed match with *MYH9* and one conserved 7-mer-m8 seed match with *ACTN4* and *VIM* in the corresponding 3′UTR. While *PLEC* and *ITGB1* have two conserved 7-mer-m8 seed matches with miR-124-3p in the 3′UTR (Figure 3B). Direct targeting of *ITGB1* and *VIM* by miR-124-3p was confirmed elsewhere (Furuta et al., 2010; Hunt et al., 2011); therefore, *ACTN4*, *MYH9*, and *PLEC* were selected for further validation.

To determine if miR-124-3p directly targets the 3′ UTR of the predicted genes, luciferase reporter plasmids were constructed containing a 400–500 bp segment of the predicted gene 3′ UTR with either the wild type or a deleted miR-124-3p seed site (Supplementary Material S3). Since miR-124-3p has two potential binding sites in the 3′UTR of *PLEC*, mutant reporter constructs were made with either single mutated sites or both sites mutated. Co-transfection of the reporter construct containing the wild-type binding sequences for the *ACTN4* 3′UTR, *PLEC* 3′UTR, and *MYH9* 3′UTR with mature miR-124-3p mimics resulted in a significant reduction in luciferase activity in neuroblastoma cells when compared to a scrambled control sequence, confirming these cytoskeletal genes as direct targets of miR-124-3p (Figure 3C).

### Confirmation of MiRNA-Target Knockdown *in vitro*

**FIGURE 4:**
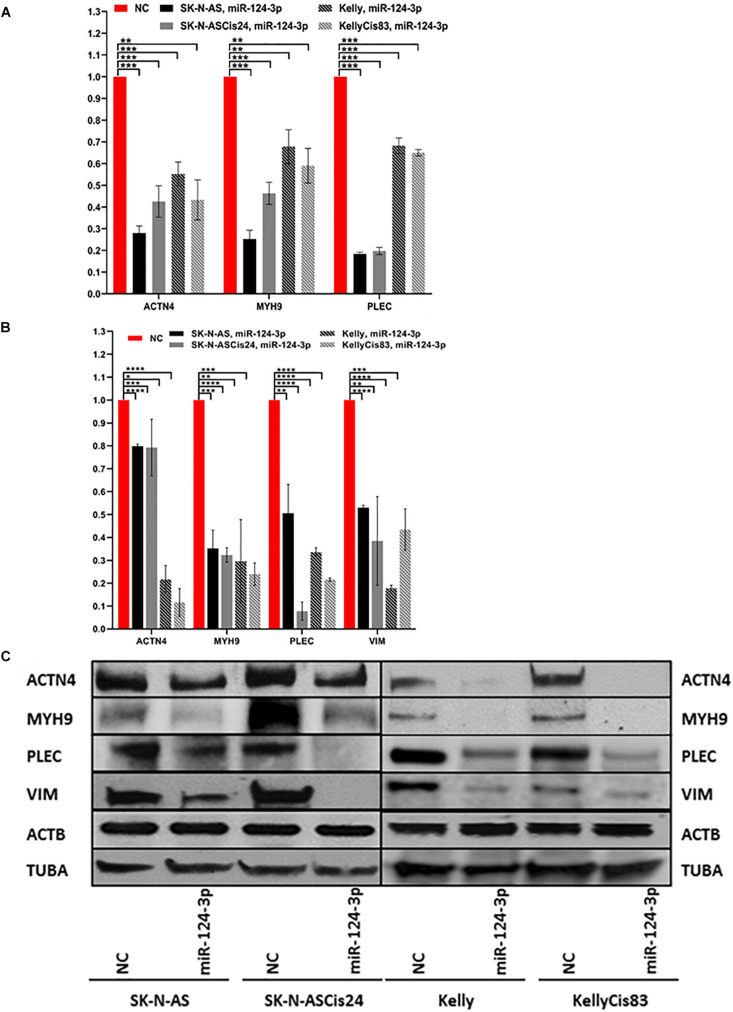
**(A)** mRNA expression of ACTN4, MYH9, and PLEC 48 h after transfection with miR-124-3p mimic or negative control in SK-N-AS/SK-N-ASCis24/Kelly/KellyCis83, determined by RT-qPCR. **(B)** Densitometry analysis of three biological repeat experiments of ACTN4, MYH9, PLEC, VIM, ACTB and TUBA expression in miR-124-3p and scrambled oligonucleotide (NC) transfected SK-N-AS/SK-N-ASCis24/Kelly/KellyCis83 neuroblastoma cell lines. **(C)** Representative western blot of target protein expression 48 h after transfection. Considering miR-124-3p targets multiple cytoskeletal genes ACTB and TUBA were used in combination as loading controls. Asterisks indicate statistical significance obtained using unpaired Student’s *t*-test (*p < 0.05, **p < 0.01, ***p < 0.001, ****p < 0.0001).

Over expression of miR-124-3p mimics resulted in consistent knockdown of cytoskeletal target genes *ACTN4*, *MYH9, VIM*, and *PLEC* in both the adrenergic Kelly/KellyCis83 and mesenchymal SK-N-AS/SK-N-ASCis24 cell lines at mRNA and protein level, in line with results from luciferase reporter assay (Figures 4A–C). Complete repression of ACTN4 protein expression relative to untreated controls was reported in Kelly/KellyCis83, which demonstrated substantially lower endogenous protein levels compared to SK-N-AS/SK-N-ASCis24. Whereas MYH9, PLEC and VIM demonstrated similar protein expression across all cell lines in response to miR-124-3p expression. The combined bioinformatics approach with functional validation identified miR-124-3p as being central to cytoskeletal remodeling and cellular transformation in our neuroblastoma models.

### MiR-124-3p Arrests Cell Cycle

**FIGURE 5:**
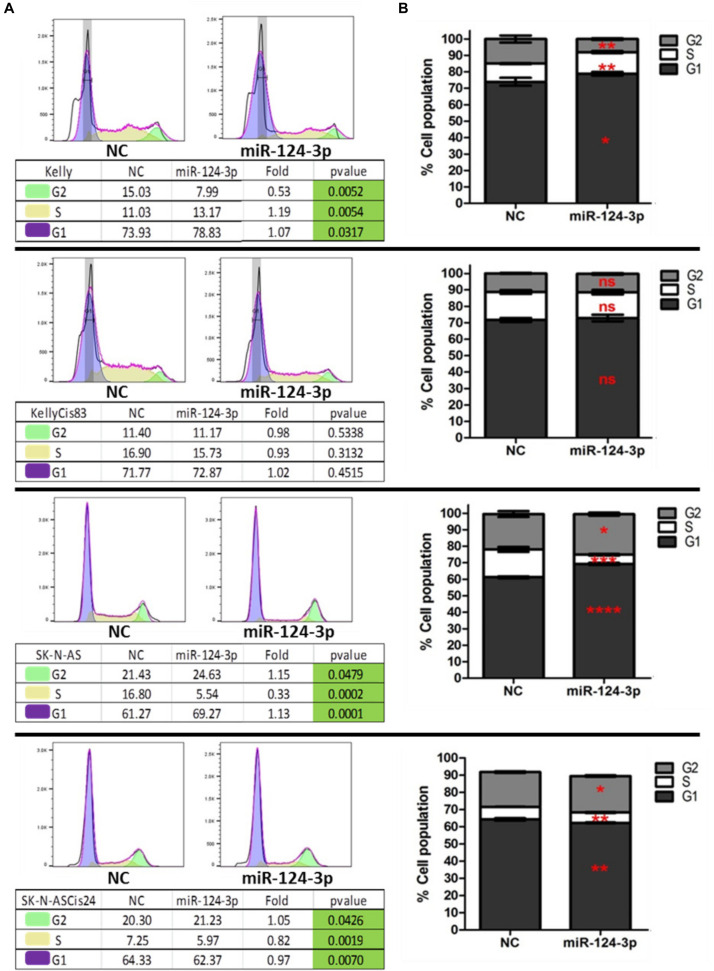
**(A)** Representative histograms of cell cycle distribution by PI staining within viable Kelly/KellyCis83 and SK-N-AS/SK-N-ASCis24 cells at 48 h and 96 h, respectively, following transfection with scrambled NC and miR-124-3p mimic. **(B)** Cell cycle distribution of PI stained Kelly/KellyCis83/SK-N-AS and SK-N-ASCis24 cells from three independent experiments. Asterisks indicate statistical significance obtained using unpaired Student’s *t*-test (*p < 0.05, **p < 0.01, ***p < 0.001, ****p < 0.0001).

In order to further investigate the cellular mechanisms associated with the suppressive effect of miR-124-3p on cell growth in neuroblastoma *in vitro*, we examined the influence of miR-124-3p expression on cell cycle progression and distribution. Analysis of the viable cell population at 48 h revealed a substantial response to miR-124-3p expression in the SK-N-AS cell line, consistent with the results obtained from cell viability assay. In SK-N-AS a 3 fold decrease in cells in the S phase from 16.80 to 5.54% (*p* = 0.0002), with a concomitant accumulation of cells in the G1 phase [61.27 to 69.27% (*p* = 0.0001)] and G2 phase [21.43 to 24.63% (*p* = 0.0479)] was detected in miR-124-3p transfected samples, indicating G1/G2 arrest and inhibition of DNA synthesis. The Kelly cell line exhibited a minor increase in cells in the G1 phase [73.93 to 78.83% (*p* = 0.0317)] and S phase [11.03 to 13.17% (*p* = 0.0054)], along with an associated decrease in cells in the G2 phase [15.03 to 7.99% (*p* = 0.0052)], suggesting a G1/S arrest slowing progression through the S phase and resulting in a lower proportion of cells in G2 phase. In contrast to the parental SK-N-AS and Kelly cell lines, their cisplatin pulse selected sub-lines SK-N-ASCis24 and KellyCis83 did not demonstrate any substantial alterations in cell cycle distribution measured at 96 h and 48 h, respectively (Figure 5). These findings validate the cell cycle modulating potential of miR-124-3p dysregulating progression through cell cycle checkpoints.

### MiR-124-3p Induces Apoptosis in Neuroblastoma Models *in vitro*

**FIGURE 6:**
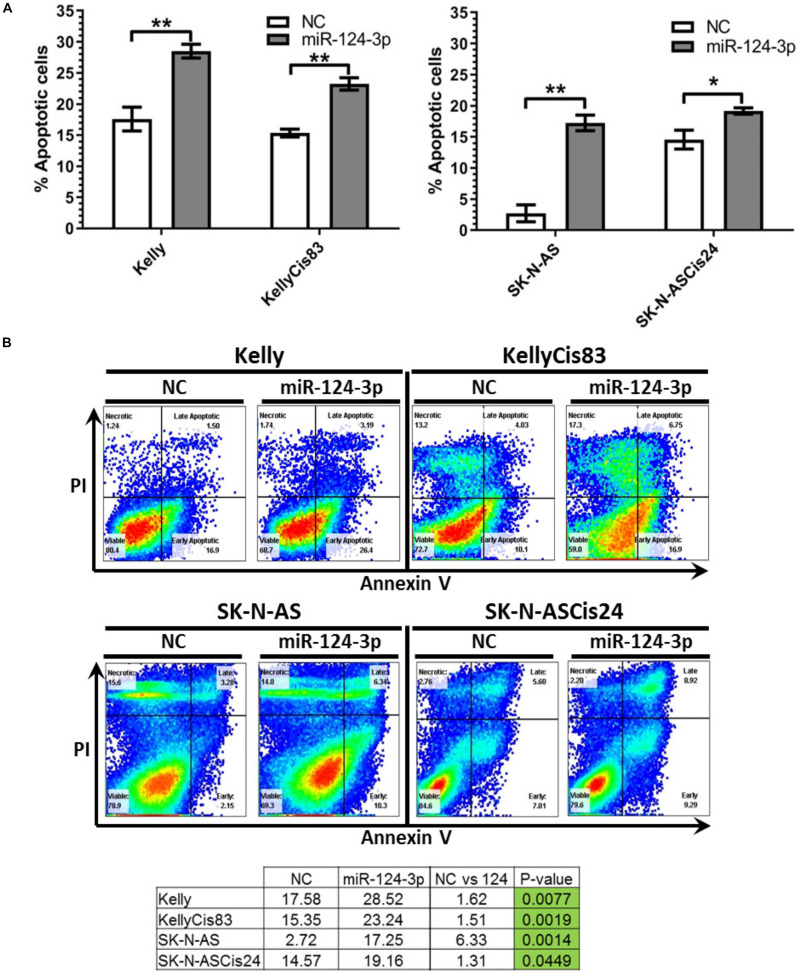
**(A)** Mean percentage of annexin V-positive Kelly/KellyCis83 and SK-N-AS/SK-N-ASCis24 cells from three independent experiments at 48 h and 96 h, respectively, following transfection with scrambled NC, miR-124-3p mimic or Kiff11. **(B)** Representative scatter plots of PI vs. Annexin V-FITC staining in one experiment with Kelly/KellyCis83 and SK-N-AS/SK-N-ASCis24. Asterisks indicate statistical significance obtained using unpaired Student’s *t*-test (*p < 0.05, **p < 0.01, ***p < 0.001, ****p < 0.0001).

To determine if the diminished proliferation and viability observed in our cell lines in response to miR-124-3p transfection were due to activation of the canonical apoptotic programmed cell death pathway, apoptosis induction was measured by flow cytometry analysis of cells stained with Annexin V and propidium iodide following miR-124-3p transfection. In order to facilitate direct comparison within cell line pair’s analysis was carried out at 48 h in the Kelly and KellyCis83 cell lines and 96 h in the SK-N-AS and SK-N-ASCis24 cell lines (Figure 6).

Transfection with miR-124-3p resulted in a significant increase in apoptotic cells from 17.58 to 28.52% (*p* = 0.0077) and 15.35 to 23.24% (*p* = 0.0025) in Kelly and KellyCis83 cell lines, respectively. The SK-N-AS cell line was analyzed 96 h following miR-124-3p expression, demonstrating the most substantial apoptosis induction of all cell lines tested with total apoptotic cells increasing substantially from 2.72 to 17.35% (*p* = 0.0014). This is consistent with the results obtained from cell viability and cell cycle analysis where SK-N-AS cells exhibited the most substantial response to miR-124-3p expression. The SK-N-ASCis24 displayed the lowest induction of apoptosis of all cell lines tested, consistent with viability and cell cycle results, exhibiting a nominal increase from 14.57 to 19.16% (*p* = 0.0449).

Taken together, these findings reveal the induction of apoptosis to highly varying degrees in all neuroblastoma cell lines tested. The proportion of cells undergoing apoptotic programmed cell death in response to miR-124-3p did not reflect the full extent of suppression of cell viability shown, thereby, demonstrating the larger combined effect of cell cycle inhibition, apoptosis and differentiation by this miRNA. The divergent activation of the apoptotic pathway between cell lines in response to miR-124-3p expression highlights their contingence on the substantially varied cell-type-specific expressional landscape of a multitude of cytoskeletal, cell cycle and apoptosis regulating genes for the functional effects of highly pleiotropic miR-124-3p.

### MiR-124-3p Resensitizes Resistant Cells to Chemotherapy

**FIGURE 7:**
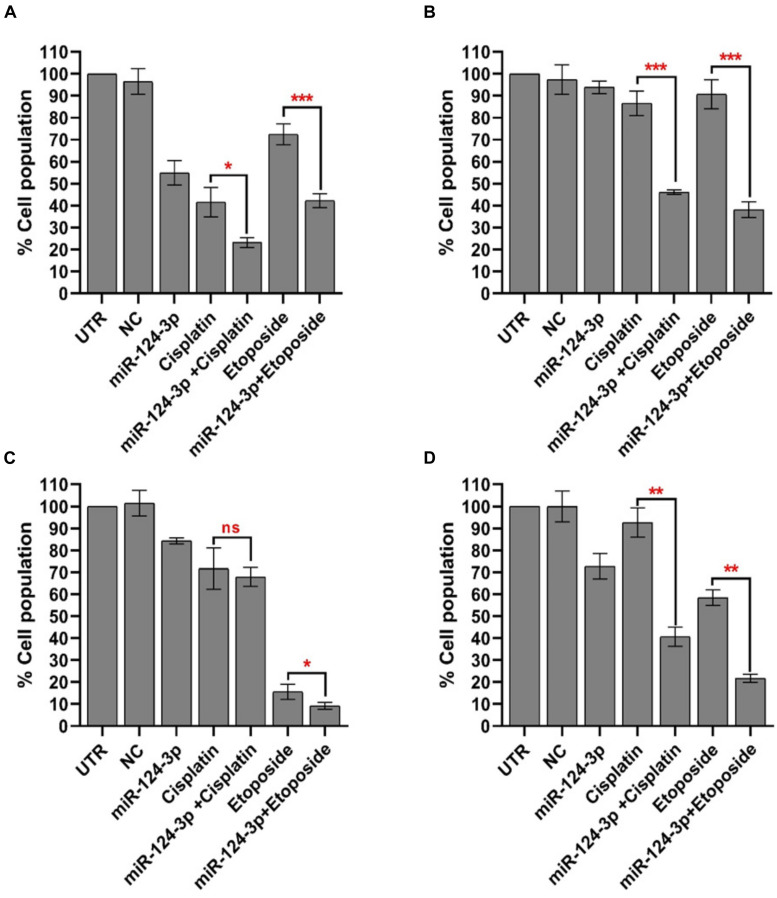
Mean percentage of viable **(A)** SK-N-AS/**(B)** SK-N-ASCis24/**(C)** Kelly/**(D)** KellyCis83 cells from three independent experiments following transfection with scrambled NC, miR-124-3p mimic or Kiff11 and treatment with cisplatin and etoposide. Asterisks indicate statistical significance obtained using unpaired Student’s *t*-test (*p < 0.05, **p < 0.01, ***p < 0.001, ****p < 0.0001).

In order to assess the potential of miR-124-3p to overcome the cisplatin-selected cross-resistance to chemotherapeutics developed in our neuroblastoma models the Kelly/KellyCis83 and SK-N-AS/SK-N-ASCis24 sensitive/resistant cell lines were treated with cisplatin and etoposide alone and in combination with miR-124-3p (Figure 7). The IC50 of the SK-N-AS/SK-N-ASCis24 cell lines for cisplatin/etoposide was 0.68 μM/0.24 μM and 3.6 μM/0.57 μM, respectively, compared to 1.4 μM/0.12 μM and 2.45 μM/0.16 μM for the Kelly/KellyCis83 cell lines. Therefore all cell lines were treated with a dose of 3 μM cisplatin and 200 nM etoposide for 72 h, alone or in combination with miR-124-3p transfection. Consistent with the results previously reported established cross resistance, the SK-N-ASCis24 cell line demonstrated the lowest sensitivity of all cell lines tested to chemotherapy treatment alone, with the most substantial sensitization to treatment in response to miR-124-3p expression. The KellyCis83 cell line exhibited a higher relative cell number following treatment with both cisplatin and etoposide compared to the parental Kelly line, consistent with its established resistance. However, in this resistant line, we report a more substantial decrease in relative cell number in response to miR-124-3p expression than the Kelly cell line, consistent with results obtained from viability assays. Of most significance, however, we also report an enhanced suppressive effect of both cisplatin and etoposide when administered in combination with miR-124-3p in all cell lines except the parental adrenergic Kelly neuroblastoma cell line, further supporting the potential role of this miRNA in sensitizing resistant cells to chemotherapeutic treatment.

### MiR-124-3p Inhibits Neuroblastoma Cell Invasion *in vitro*

**FIGURE 8:**
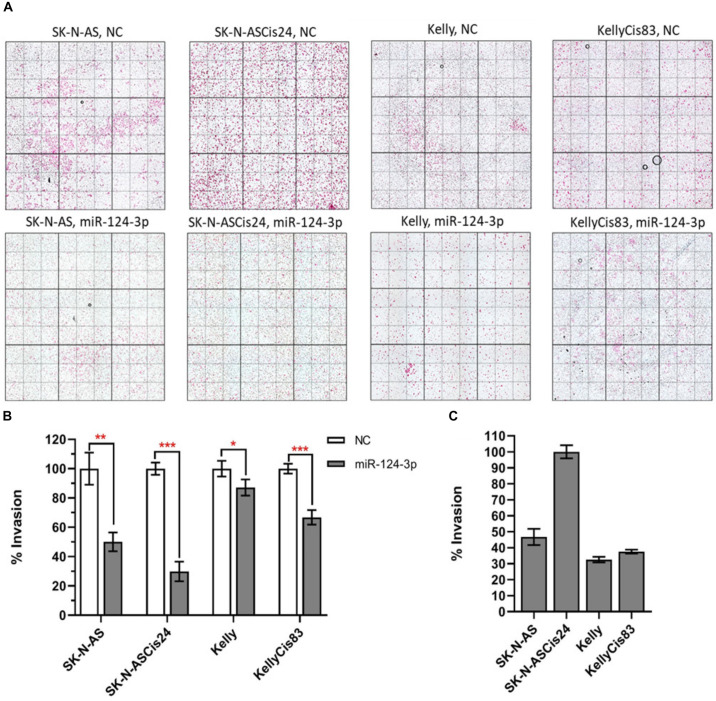
**(A)** Representative images from invasion assays for SK-N-AS, SK-N-ASCis24, Kelly, and KellyCis83 cells from three independent experiments following transfection with scrambled NC or miR-124-3p mimic. **(B)** Mean invaded cells from each cell line 48 h after transfection with miR-124-3p, as a percentage of corresponding cell lines transfected with negative control scrambled oligonucleotide. **(C)** Comparison of endogenous invasive capability of untreated sensitive and resistant adrenergic and mesenchymal neuroblastoma cell lines. Asterisks indicate statistical significance obtained using unpaired Student’s *t*-test (*p < 0.05, **p < 0.01, ***p < 0.001, ****p < 0.0001).

Over expression of miR-124-3p mimics resulted in significant knockdown of cell invasion in SK-N-AS/SK-N-ASCis24 by approx. 50% and 70%, respectively, while the Kelly/KellyCis83 cell lines demonstrated a more moderate decrease in cell invasion by approx. 10% and 30% compared to scrambled oligonucleotide transfected cells (Figures 8A,B). The invasive capability was also assessed in each neuroblastoma without treatment, demonstrating the highest invasion in the drug resistant mesenchymal SK-N-ASCis24 neuroblastoma cell line, approx. 2 fold higher than its parental cell line SK-N-AS and 2.5 fold higher than the adrenergic Kelly and KellyCis83 cell lines (Figure 8C). These findings demonstrate the enhanced invasive potential of the mesenchymal neuroblastoma cells relative to adrenergic neuroblastoma cells and a more substantial repression of this invasive potential by miR-124-3p expression in this cell type.

### Analysis of MiR-124-3p Validated Target Genes

**FIGURE 9:**
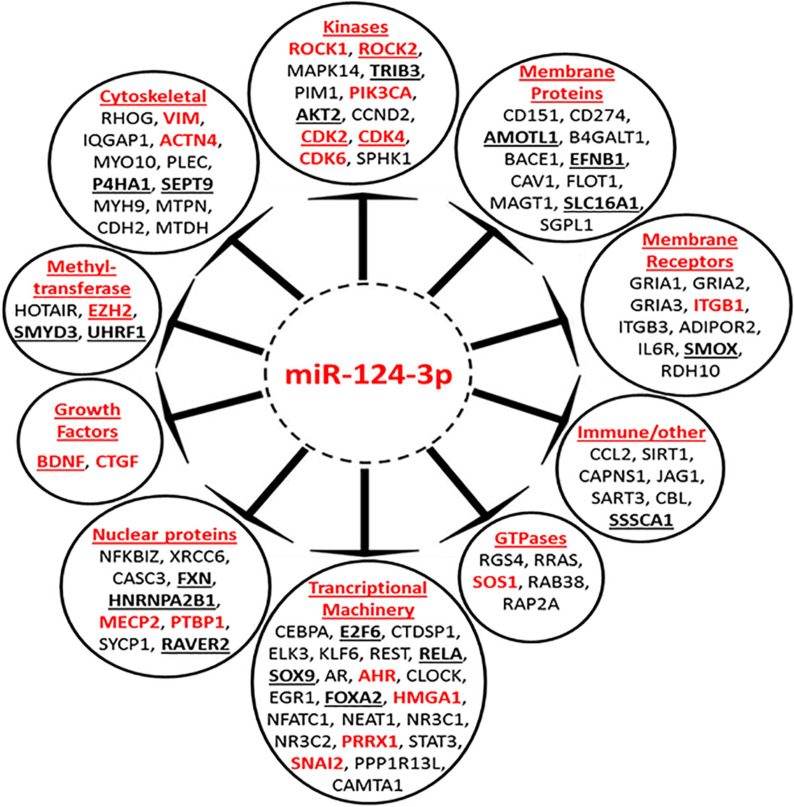
Mir-124-3p direct targets, validated by luciferase reporter assay. Genes in red font have previously been reported to be associated with neuroblastoma. High expression of underlined genes in bold is predictive of poor overall survival in a mixed neuroblastoma cohort. Genes are grouped according to their function.

To gain a better understanding of the broader biological processes regulated and influenced by miR-124-3p, a MiRTarBase search was carried out to identify the larger panel of genes targeted by this miRNA. The MiRTarBase^1^ has an extensive collection of more than 51,000 manually curated miRNA-target interactions; only targets which were validated by luciferase reporter assays were included in this study. A total of 90 luciferase reporter assay validated targets, including those from our study, were identified for miR-124-3p. A PubMed literature search was performed for references to neuroblastoma and at least one of the validated target genes, in order to select targets that were previously reported in the literature to be associated with neuroblastoma. The clinical relevance of miR-124-3p validated targets was assessed in a cohort of 276 neuroblastoma tumors of the mixed stage using the R2 database (AMC, 0000). This analysis identified 33 of 90 validated miR-124-3p targets to be associated with poor overall survival in neuroblastoma (Supplementary Material S4). Of the 33 clinically significant target genes, high expression of 23 oncogenes and low expression of 10 tumor suppressor genes were associated with poor overall survival (Supplementary Material S5). The miR-124-3p validated target genes belong to a number of functional groups including; cytoskeletal, kinase, membrane protein, membrane receptors, methyl-transferases, transcriptional machinery, GTPases, nuclear proteins and growth factors among others, emphasizing the pleiotropic and regulatory role this miRNA has in cytoskeletal organization, proliferation and motility (Figure 9). These findings substantiate the widespread influence of miR-124-3p across a range of cellular processes, interacting at different levels in various cellular pathways in a context dependant manner.

## Discussion

Neuroblastoma is characterized by its highly heterogeneous cellular composition and divergent clinical outcome. This heterogeneity in NB cell lines was first defined by the Biedler lab with the isolation of morphologically distinct N-type (neuroblastic/neuronal) sympathoadrenal neuroblasts, S-type (schwannian/non-neuronal), and I-type (intermediate) stem-like cells featuring both N- and S-type cells characteristics. These NB cell lines had distinct phenotypes and gene expression profiles and were capable of interconversion. Tumors are comprised these morphologically distinct cancer cells in varying proportions (Biedler et al., 1973; Ciccarone et al., 1989; Ross et al., 2003, 2015).

More recent studies by van Groningen et al. (2017) have further characterized and refined these cell types, dissecting the epigenetic landscape, super-enhancer and gene regulatory networks of these distinct phenotypes and describing them as adrenergic and mesenchymal neuroblastoma cells. These cell types share characteristics with the N-type and the S-type cells previously described by Biedler et al. (1973) These studies reported that both adrenergic (ADRN) and mesenchymal (MES) were tumorigenic, although adrenergic cells formed more aggressive than mesenchymal *in vivo*. The adrenergic cell-type was also reported to be more sensitive to chemotherapy than the mesenchymal cells, with enrichment of these mesenchymal cells in relapsed post-treatment samples (Singh et al., 2004; Ricci-Vitiani et al., 2007; Boeva et al., 2017; van Groningen et al., 2017, 2019; Szemes et al., 2019). This facet of neuroblastoma makes it extremely challenging to achieve sustained treatment success as different cellular subtypes which comprise tumors undergo highly varied response to chemotherapeutic intervention and differentiation treatment.

Our study found significantly higher endogenous expression of MES morphological markers, VIM and ACTN4 along with a significantly lower endogenous expression of the ADRN morphological markers NEFL, GAP43, CgA, and TUBB3 in SK-N-AS compared to Kelly, consistent with their classification by Biedler et al. (1973) and Upton et al. (2020) who reported that SK-N-AS contained super-enhancers from both cell types, while Kelly contain predominantly adrenergic super-enhancers. Pulse selection with cisplatin in these cell lines resulted in a significant up-regulation of VIM in SK-N-ASCis24 and downregulation of NEFL, CgA, and GAP43; however, the KellyCis83 demonstrated increased expression of CgA and GAP43, along with a substantial decrease in the expression of VIM (Supplementary Material S7), suggesting a phenotypic divergence of the cell lines in response to chemotherapeutic treatment, supporting the commitment of adrenergic-type neuroblastoma cells to the adrenergic lineage as reported by van Groningen et al. (2019). In the cisplatin pulse selected mesenchymal SK-N-ASCis24, we previously reported slowing of proliferation, along with a transition to a more pronounced mesenchymal morphology and senescence-like phenotype. These findings are consistent with a study by Chang et al. (1999a, b) which described the development of a drug-induced senescence-like phenotype across a panel of cell lines following prolonged exposure to doxorubicin. Subsequent studies also reported the development of a senescence-like phenotype in neuroblastoma cells after continued BrdU and hydroxyurea treatment (Narath et al., 2007; Acosta et al., 2009). The adrenergic KellyCis83 cell line, in contrast, demonstrated an increased proliferative rate following cisplatin pulse selection, further supporting the divergent response and resistance mechanism between these different cell types. This is consistent with previous reports where the mesenchymal neuroblastoma cells have a higher propensity for senescence induction than adrenergic cells (van Groningen et al., 2017). A study by Samaraweera et al. (2014) assessed miRNA expression across a panel of 13 neuroblastoma cell lines of different sub-type, reporting highest levels of miR-124-3p in adrenergic N-type cells, with 12.5 fold lower expression in stem cell-like I-type cells and barely detectable levels in mesenchymal S-type cells. The same study also demonstrated miR-124-3p induced terminal differentiation of highly tumorigenic stem cells to less tumorigenic adrenergic phenotype, supporting its role in suppressing neuroblastoma progression. In line with these previous reports, our study confirmed a higher endogenous expression of miR-124-3p in the adrenergic Kelly/KellyCis83 cell lines compared to the mesenchymal lines SK-N-AS/SK-N-ASCis24, with a demonstrated ∼50% higher expression in the chemo sensitive parental cell lines of both type than their cisplatin selected resistant sub-lines.

The role of miR-124-3p in influencing the cell fate and phenotype has been well established in the literature; however, many of these publications do not emphasize the major influence of cell-type context in the activity of this miRNA. MiR-124-3p is highly conserved and expressed in the brain at >100 times the levels in other tissues, highlighting its neuronal specificity (Lagos-Quintana et al., 2002; Sun et al., 2015). This miRNA is expressed at very low levels in neural progenitor cells but highly expressed in differentiating and mature neurons. It plays an important role in maintaining neuronal cell identity during neurogenesis, promoting neuronal properties while suppressing progenitor functions and non-neuronal transcripts as neural progenitors develop into mature neurons (Lim et al., 2005; Deo et al., 2006; Cao et al., 2007). In the case of neuroblastoma, this miRNA has been repeatedly demonstrated to have a modest but integral role in driving the differentiation of neuroblast cells. Cao et al. (2007) described a role for miR-124-3p in determining neuronal differentiation, through knockdown of neural progenitor genes, laminin γ1 (*LAMC1*) and integrin β1 (*ITGB1*). Knock-down experiments of miR-124-3p were previously reported to cause defects in adult neurogenesis, highlighting miR-124-3p as an attractive candidate for differentiation induction therapeutics in particular cell types (Cheng et al., 2009; Clark et al., 2010).

Our study identified and validated *MYH9*, *ACTN4* and *PLEC* as direct targets of the clinically relevant neuronal miR-124-3p. These cytoskeletal genes (*MYH9*, *PLEC, ITGB1*) and mesenchymal markers (*VIM*, *ACTN4*), were previously reported by our lab to be up-regulated with cisplatin resistance development and involved in phenotypic and morphological modulation observed in our neuroblastoma cell model (Piskareva et al., 2015). We demonstrated miR-124-3p mediated knockdown *in vitro* of these cytoskeletal genes, inducing morphological reversion toward more epithelial-like morphology, from cells with a mesenchymal resistant neuroblastoma cell phenotype. The non-muscle myosin II (*MYH9*) is a conventional motor protein which generates intracellular contractile forces and tension as well as driving cell spreading, migration, cytokinesis, and morphogenesis (Guha et al., 2005; Murthy and Wadsworth, 2005; Vicente-Manzanares et al., 2009; Aguilar-Cuenca et al., 2014). MYH9 expression is associated with poorer differentiation and prognosis, intratumoral vascular and lymphatic invasion in resected non-small cell lung cancer, and knockdown by let-7f resulted in suppression of invasion and metastasis in gastric cancer (Liang et al., 2011). Plectin (*PLEC*) is a cytoskeletal linker protein of the plakin family which regulates cytoskeleton dynamics and mechanically stabilizes the cell by connecting intermediate filaments with desmosomes and hemidesmosomes. High expression of PLEC is associated with poor prognosis in breast and colorectal cancer and was reported to drive cancer metastasis, by stabilizing invadopodia via anchoring to vimentin intermediate filament scaffolds, a necessary process in cancer cell invasion and extravasation (Chao et al., 1996; Lu et al., 2008; Sutoh Yoneyama et al., 2014). Integrin β1 (*ITGB1*) is an integrin family transmembrane receptor protein which binds the extracellular matrix and controls cell shape and cytoskeletal structure in response to extracellular forces, highlighting its role in migration and invasion. Further support for the tumor suppressive role of miR-124-3p was provided by Hunt et al. (2011) who reported decreased migration and invasion in oral squamous cell carcinoma, following miR-124-3p knockdown of *ITGB1*. Vimentin (*VIM*) is central to cytoskeletal modulation associated with EMT and a key determinant of non-neuronal morphology. As cells undergo morphological change, intermediate filaments transform from a keratin-rich network, connecting to adherens junctions, to a vimentin rich network connecting to focal adhesions (Kokkinos et al., 2007). High expression of VIM was found in esophageal squamous cell carcinoma and non-small cell lung cancer tumors with much more advanced tumor status and a higher incidence of lymph node metastasis (Jin et al., 2010; Dauphin et al., 2013). Alpha-actinin 4 (*ACTN4*) is a key component of the cytoplasmic surface of cell adhesion sites, including focal adhesions and adherens junctions. Increased expression was reported to suppress focal adhesion maturation resulting in invasive growth and lymphatic spread of cancer cells in colorectal, bladder, and ovarian clear-cell adenocarcinoma (Honda et al., 2005; Barbolina et al., 2008; Yamamoto et al., 2012; Yoshii et al., 2013; Fukumoto et al., 2015). The results obtained from our study support the modulation of essential cytoskeletal genes by miR-124-3p and the influence of this miRNA on cytoskeletal structure, cell morphology and invasive capability. In the adrenergic Kelly and KellyCis83 cell lines, where endogenous expression of the mesenchymal markers and cytoskeletal genes is very low, miR-124-3p expression drives neuronal differentiation of cells, through direct targeting of key cytoskeletal genes instead of activation of canonical epithelial to mesenchymal transition or differentiation pathways.

Multiple studies have reported a tumor suppressive role of miR-124-3p across many cancers including neuroblastoma, medulloblastoma (Li et al., 2009; Silber et al., 2013), glioblastoma (Silber et al., 2008; Chen et al., 2015), hepatocellular carcinoma (Furuta et al., 2010), prostate, breast (Feng et al., 2015; Wang et al., 2016), gastric (Xia et al., 2012; Xie et al., 2014), colorectal (Wang et al., 2013; Zhou et al., 2016), and non-small cell lung cancer (Lin et al., 2016). Although the involvement of miR-124-3p in proliferation, invasion and apoptosis has been demonstrated across a range of cancers, the current knowledge of its effect in neuroblastoma is limited, with reports of knockdown of miR-124-3p in SK-N-SH neuroblastoma cells inducing differentiation, cell cycle arrest and apoptosis through promoting the aryl hydrocarbon receptor (AHR) (Huang et al., 2011). However, the vast majority of studies report a tumor suppressive role for this miRNA, with a varied context and cancer dependant effect, such as the study by Le et al. (2009) where up-regulation of miR-124-3p was detected in differentiated SH-SY5Y neuroblastoma cells and shown to increase differentiated cells in response to ectopic overexpression. Our study confirmed the cell viability suppressive role of miR-124-3p across different neuroblastoma cell sub-types in addition to its potential as an inhibitor of cell proliferation and invasion, a chemosensitizer and an apoptosis inducer *in vitro*.

The G1 cell cycle arrest detected in the Kelly and SK-N-AS cell lines following miR-124-3p expression further supports previous reports of miR-124-3p inhibition of proliferation in a range of hepatocellular carcinoma cell lines, decreasing cells in S and G2/M phase and increasing cells in G0/G1 phase (Furuta et al., 2010). This subtle and specific influence of miR-124-3p on cell cycle progression is also consistent with GO analysis which found target genes to be involved in a limited number of specific cell cycle stages such as “Mitotic G1-G1/S Phases,” rather than targeting the pathway as a whole. Similarly, when assessing the apoptosis induction in response to miR-124-3p expression, rather than observing a strong level of apoptosis induction, a more modest increase was detected in the Kelly/KellyCis83 and SK-N-AS. This finding is in agreement with our GO study of miR-124-3p validated targets, where genes were found to be involved exclusively in a few downstream stages of the Programmed cell death pathway including the “Apoptotic Execution Phase,” rather than acting upstream to activate the apoptotic pathway.

A substantially lower induction of apoptosis and G1 cell cycle arrest in response to miR-124-3p expression in SK-N-ASCis24 is most likely due to its slower proliferation, deletion of p53 and more senescent phenotype, with only minimal increase in early apoptosis as cells exhibit a slow downstream response to cell cycle arrest and apoptosis induction following miR-124-3p transfection. This is consistent with the senescent state of this cell line which can act as resistant non-proliferating tumors cells capable of supporting and driving the growth of surrounding cells (Coppola et al., 2014; Ritschka et al., 2017). A recent study by Milanovic et al. (2018) described an oncogenic role of senescent cells in the tumor cell population, with senescence-associated reprogramming of cells promoting cancer stemness and driving aggressive cell growth upon escape from the cell cycle arrest with enrichment of these reprogrammed cells found within relapse tumors. These reports emphasize the importance of this cell state in determining disease outcome and implicate miR-124-3p in this process.

In addition to demonstrating miR-124-3p targeting of key cytoskeletal genes, modulation of cell morphology, perturbation of cell cycle progression, and induction of apoptosis to varying degrees across different sensitive and resistant neuroblastoma cell-types, our study has also shown substantial increase in sensitivity to chemotherapy treatment in cross resistant cell lines of both adrenergic and mesenchymal cell-type through miR-124-3p expression. Finally, this miRNA was also found to substantially inhibit the enhanced invasive capacity of our drug-resistant mesenchymal SK-N-ASCis24 neuroblastoma cell line *in vitro*, supporting the role of miR-124-3p in reversing resistance associated cellular processes within different cellular contexts.

## Materials and Methods

### Cell Lines

The Kelly cell line is *MYCN* amplified with a 17q chromosomal gain. The SK-N-AS cell line is *MYCN* diploid cell and derived from a metastatic bone marrow site of INSS stage 4 disease of an 8-year-old female. It has a deletion at chromosome 1p and 11q as well as a gain at 17q. The Kelly and SK-N-AS cell lines were obtained from the European Cell Culture Collection. The chemotherapy-resistant sub-lines were developed by exposing cells to increasing concentrations of cisplatin over 6 months as described for the SK-N-AS/SK-N-ASCis24 sensitive/resistant pair in Harvey et al. (2015) and comprehensively characterized in Piskareva et al. (2015). Cells used for this study were taken from frozen stocks of these resistant sub-lines. Resistance was confirmed by toxicity assay, and experiments were conducted within seven passages from thawing. KellyCis83 demonstrated a 1.75 fold (*p* = 0.0001) increase in cisplatin IC50 compared to parental cell line while SK-N-ASCis24 had a substantial 5.3 fold (*p* = 0.00002) increase (Piskareva et al., 2015). Developed cell lines also demonstrated cross-resistance to Etoposide (KellyCis83, 1.33 fold, *p* = 0.004, SK-N-ASCis24, 2.25 fold, *p* = 0.004) and Irinotecan (KellyCis83, two-fold, *p* = 0.002, SK-N-ASCis24, 5.37 fold, *p* = 0.007). Cells were routinely screened for mycoplasma using MycoAlert Mycoplasma Detection kit (Lonza, #LT07-318). Cell lines were authenticated by STR PCR (SOP ECACC/047). Kelly/KellyCis83 cells were cultured in RPMI (Gibco, #21875-034), 10% Fetal Bovine Serum (Gibco, # 10270106), 1% Penicillin/Streptomycin (Gibco, #15070). SKN-AS/SK-N-ASCis24 cells were cultured in MEM (Gibco, #21090-022), 1% Non-essential Amino Acids (Gibco, #11140-050), 200 mM Glutamine (Gibco, #25030-024), 10% Fetal Bovine Serum (Gibco, #10270106), 1% Penicillin/Streptomycin (Gibco, #15070). All cell lines were incubated at 37°C in a humidified chamber with 5% CO_2_.

### Transfection Procedures

MiR-124-3p miRNA mimic (Ambion, MC10691), positive (Ambion, S7902), and scrambled negative oligonucleotide controls (Ambion, AM17110) were transiently transfected into cells at a final concentration of 10 nM using Lipofectamine^®^ RNAiMAX (Invitrogen, 13775-150) as per manufacturer’s instructions (Nolan et al., 2017). Transfections were performed in 96-well plate for cell viability assay or 6-well for miRNA/gene expression, protein expression, cell morphology, apoptosis, and cell cycle assessment.

### Luciferase Reporter Assay

Direct targeting of the ACTN4 (NM_004924), PLEC (NM_ 000445), and MYH9 (NM_002473) 3′UTRs was determined by cloning of the corresponding 3′UTR seed region and mutated seed regions into separate psiCHECK^TM^-2 vectors (Eurofins MWG Operon, Anzinger Str., Ebersberg, Germany). Renilla and firefly luciferase activities were measured using the Dual-Luciferase^®^ Reporter kit (Promega) and luminescence recorded on a Victor X3 2030 Multilabel Plate Reader (PerkinElmer). Results were reported as mean S.E.M and differences were tested for significance using 2-tailed Students *t*-test.

### Quantitative Real-Time RT-PCR

Total RNA was extracted from cell lines using miRNeasy Mini Kits (Qiagen, Valencia, CA, United States). Reverse transcription was performed using total RNA with primers specific for miR-124-3p or RNU48 control and TaqMan microRNA reverse transcription kit (Applied Biosystems Life Technologies, Carlsbad, CA, United States). For gene expression analysis, reverse transcription was performed using High-Capacity reverse transcription kits (Applied Biosystems). Specific TaqMan assays (Applied Biosystems) for ACTN4, MYH9 and were employed for expression analysis on the 7900 HT Fast Real-Time System (Applied Biosystems). MiRNA and gene expression were normalized using the endogenous controls RNU48 and 18S, respectively, and relative quantities determined by the delta CT method (Mestdagh et al., 2009).

### Western Blot Analysis

Total protein was analyzed by western blotting using primary antibodies anti-ACTN4 (ab32816), ant-PLEC (ab32528), anti-MYH9 (ab55456), anti-alpha-tubulin (7291), anti-beta-actin (ab6276), and anti-VIM (ab71144), followed by anti-mouse (ab6728) or anti-rabbit (ab97200) secondary antibody and anti-mouse alpha-tubulin loading control (Abcam, Cambridge, MA, United States).

### Cell Viability Assay

Cells were seeded at 10^4^ cells/mL suspension in 96-well plates at 100 μL/well and incubated overnight at 37°C in 5% CO2, before transfection. Alternatively, cells were seeded at 2 × 10^4^ cells/mL suspension in 96-well plates at 100 μL/well and incubated overnight at 37°C in 5% CO2, prior to treatment with 3 μM Cisplatin (Hospira UK Ltd., #PA437/4/7) and 200 nM Etoposide (Ebewe Pharma, #pa789/13/1) (Piskareva et al., 2015). Assessment of cell viability was determined at 72 h post-chemotherapy or every 24 h from 0 to 120 h or 168 h following transfection by acid phosphatase assay as previously described (Martin and Clynes, 1993; Harvey et al., 2015) and quantified on a VICTOR^TM^ X dual-beam plate reader (Perkin Elmer) at 405 nm with a reference wavelength of 620 nm.

### Cell Cycle Analysis

Cell cycle progression and proliferation were monitored using propidium iodide staining at 48 h and 96 h post-transfection (Abcam, Cambridge, MA, United States). Viable cells were acquired using a BD FACSCanto II flow cytometer (Becton Dickinson, San Jose, CA, United States) and BD FACSDIVA software.

### Invasion Assay

Invasion assays were carried out using BD BioCoat^TM^ Growth Factor Reduced MATRIGEL^TM^ Invasion Chamber as per manufacturers’ instructions (BD Biosciences, San Jose, CA, United States). To determine the average number of invading cells, inserts were then stained with crystal violet and viewed under the Nikon Eclipse 90i microscope and the number of invaded cells in 9 fields, were counted at 100× magnification. Mean values of triplicate experiments were calculated and results subjected to *t*-test.

### Apoptosis Analysis

Apoptosis levels were demonstrated by flow cytometric analysis using the Tali^®^ Apoptosis Kit – Annexin V Alexa Fluor^®^ 488 & Propidium Iodide (Thermo Scientific, #A10788). Cells were acquired using a BD FACSCanto II flow cytometer (Becton Dickinson, San Jose, CA, United States) and BD FACSDIVA^TM^ software.

### R2 Database

The web-based database contains data submitted by multiple research groups^2^. The application was developed by the department of oncogenomics^3^ in the Academic Medical Center (AMC) and allowed for the visualization of the submitted microarray data from gene and miRNA expression arrays. Data was compiled from tumor sets including; from Dublin, Ghent, Essen, and Genoa.

### Statistics

All statistical analysis was performed using GraphPad Prism 5 software (GraphPad Software). Statistical significance was determined for all experimental data by using the unpaired Student’s *t*-test. In all cases, error bars are representative of the standard deviation of the mean of three biological experiments unless otherwise stated. A *P*-value of <0.05; ^∗∗^*P* < 0.01; ^∗∗∗^*P* < 0.001).

## Conclusion

MiR-124-3p is a regulator of the neuronal cell phenotype which has been demonstrated in the literature to be integral to neurogenesis and neuronal cell identity. While it has been widely reported to have a tumor suppressive effect in a variety of cancers, the role of miR-124-3p in neuroblastoma is not as clear. As a result of the highly varied epigenetic and phenotypic landscape of cells which comprise neuroblastoma the effect of this miRNA within neuroblastoma is highly contingent on the cell type in which it is acting. Our study demonstrates direct targeting and knockdown by miR-124-3p of a panel of key cytoskeletal genes upregulated with resistance development and highlights the variation in response to miR-124-3p between different cellular phenotypes. We demonstrated the ability of this miRNA to influence the differentiation state of cells and modulate the progression of cells through the cell cycle. Finally, this miRNA was demonstrated to induce mild apoptosis, sensitize chemotherapy-resistant cells to treatment and inhibit cell invasive capability. This study highlights the potential of miR-124-3p as a potential candidate for incorporation into a panel of therapeutic miRNA by retaining cells in a state of sensitivity to chemotherapeutics or driving differentiation of cells to mature neurons.

## Data Availability Statement

The datasets presented in this study can be found in online repositories. The names of the repository/repositories and accession number(s) can be found in the article/Supplementary Material.

## Author Contributions

JN and OP: conceptualization and writing – original draft. JN, SC, MS, AB, MFS, and OP: data curation. JN, MS, AB, MFS, JP, and OP: formal analysis. RS and OP: funding acquisition. JN, SC, MS, JF, AB, RS, and OP: investigation. JN, SC, AB, MFS, JP, RS, and OP: methodology. OP: project administration. MFS, JP, and RS: resources. MS, AB, and JP: software. JP and OP: supervision. JN, SC, and JF: validation. JN, MS, JF, AB, and OP: visualization. JN, MS, AB, MFS, JP, RS, and OP: writing – review and editing. All authors contributed to the article and approved the submitted version.

## Conflict of Interest

The authors declare that the research was conducted in the absence of any commercial or financial relationships that could be construed as a potential conflict of interest.

## Footnotes

1 mirtarbase.mbc.nctu.edu.tw

2 http://hgserver1.amc.nl/cgi-bin/r2/main.cgi

3 https://r2-faqs-workflows.readthedocs.io/en/latest/
