# Supplementary material for: A Context-Dependent Role for MiR-124-3p on Cell Phenotype, Viability and Chemosensitivity in Neuroblastoma in vitro
Source: Front Cell Dev Biol. 2020 Nov 20;8:559553. doi: 10.3389/fcell.2020.559553 (PMC7714770; doi:10.3389/fcell.2020.559553)
Supplement: Supplementary file 1 [file Presentation_1.pptx]

## Slide 1
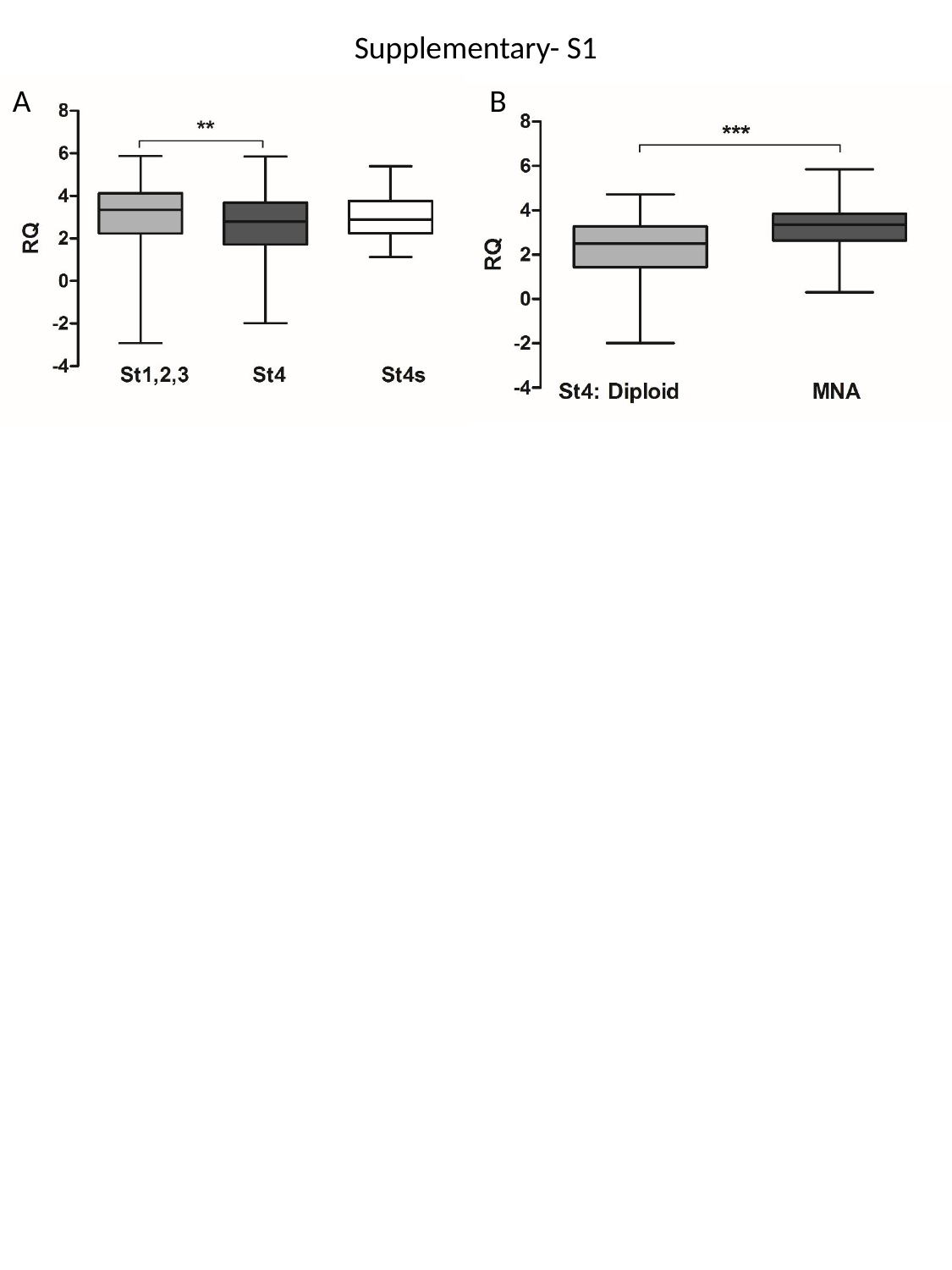

Supplementary- S1
A
B

## Slide 2
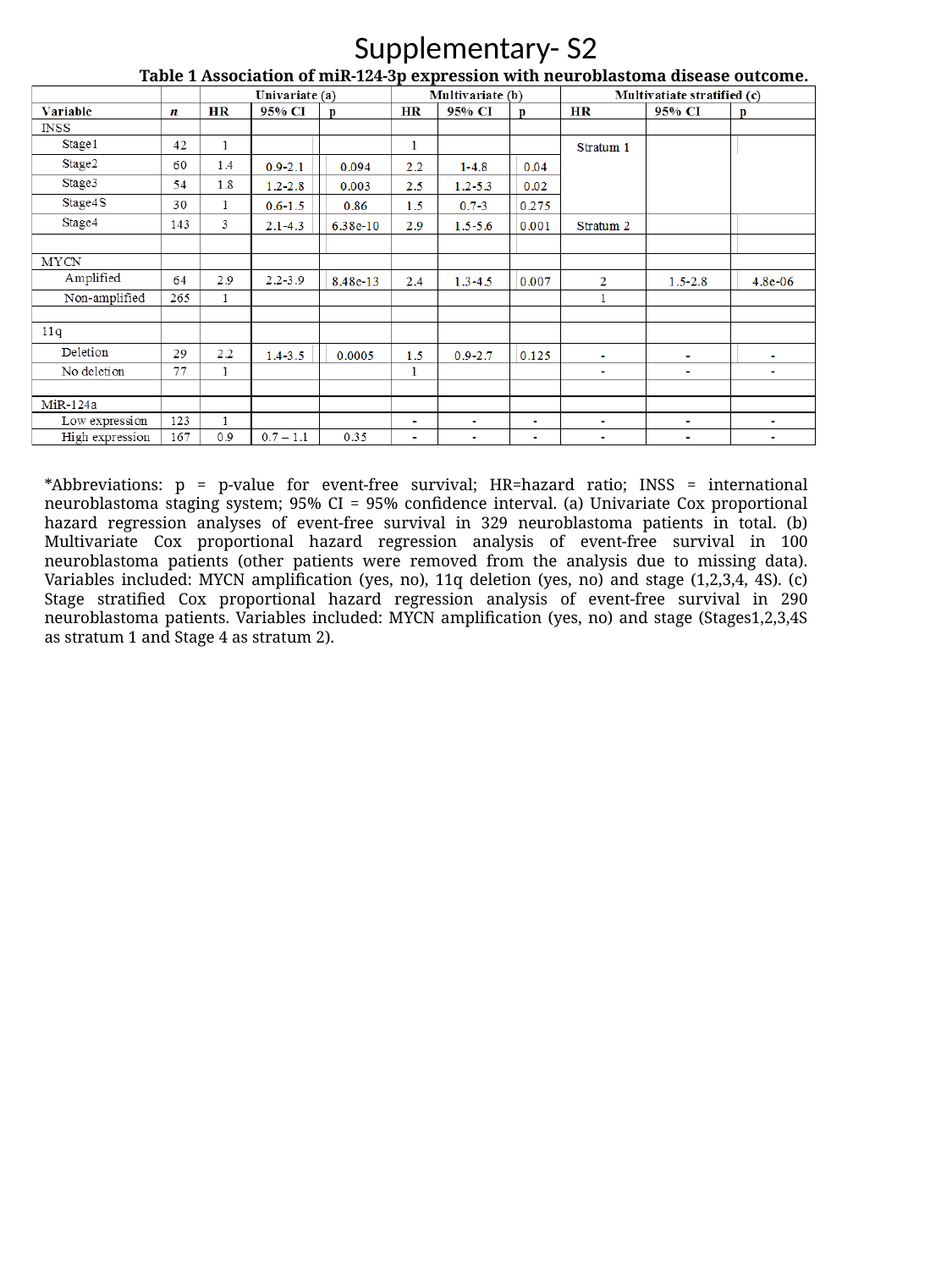

Supplementary- S2
Table 1 Association of miR-124-3p expression with neuroblastoma disease outcome.
*Abbreviations: p = p-value for event-free survival; HR=hazard ratio; INSS = international neuroblastoma staging system; 95% CI = 95% confidence interval. (a) Univariate Cox proportional hazard regression analyses of event-free survival in 329 neuroblastoma patients in total. (b) Multivariate Cox proportional hazard regression analysis of event-free survival in 100 neuroblastoma patients (other patients were removed from the analysis due to missing data). Variables included: MYCN amplification (yes, no), 11q deletion (yes, no) and stage (1,2,3,4, 4S). (c) Stage stratified Cox proportional hazard regression analysis of event-free survival in 290 neuroblastoma patients. Variables included: MYCN amplification (yes, no) and stage (Stages1,2,3,4S as stratum 1 and Stage 4 as stratum 2).

## Slide 3
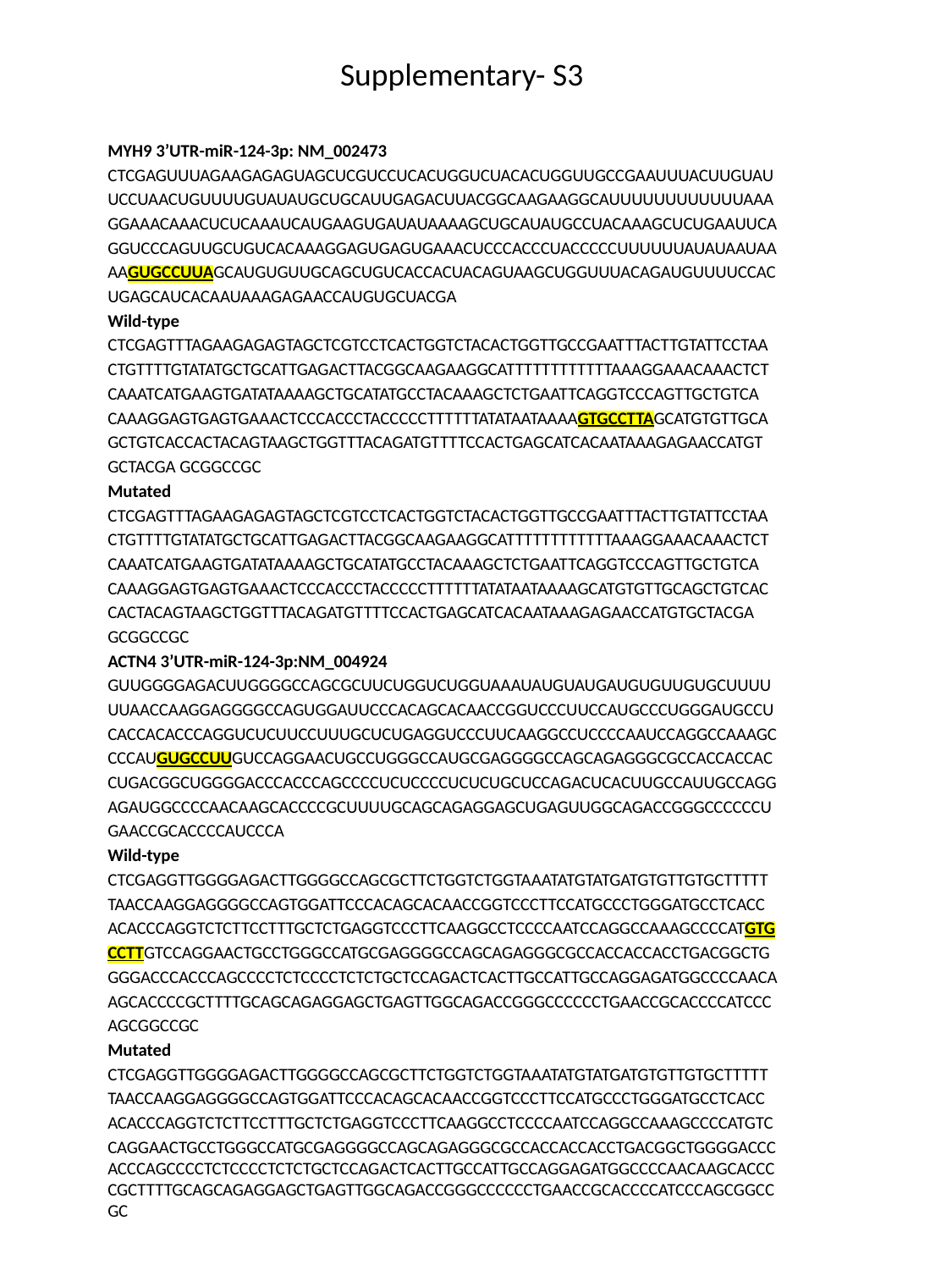

Supplementary- S3
MYH9 3’UTR-miR-124-3p: NM_002473
CTCGAGUUUAGAAGAGAGUAGCUCGUCCUCACUGGUCUACACUGGUUGCCGAAUUUACUUGUAU
UCCUAACUGUUUUGUAUAUGCUGCAUUGAGACUUACGGCAAGAAGGCAUUUUUUUUUUUUAAA
GGAAACAAACUCUCAAAUCAUGAAGUGAUAUAAAAGCUGCAUAUGCCUACAAAGCUCUGAAUUCA
GGUCCCAGUUGCUGUCACAAAGGAGUGAGUGAAACUCCCACCCUACCCCCUUUUUUAUAUAAUAA
AAGUGCCUUAGCAUGUGUUGCAGCUGUCACCACUACAGUAAGCUGGUUUACAGAUGUUUUCCAC
UGAGCAUCACAAUAAAGAGAACCAUGUGCUACGA
Wild-type
CTCGAGTTTAGAAGAGAGTAGCTCGTCCTCACTGGTCTACACTGGTTGCCGAATTTACTTGTATTCCTAA
CTGTTTTGTATATGCTGCATTGAGACTTACGGCAAGAAGGCATTTTTTTTTTTTAAAGGAAACAAACTCT
CAAATCATGAAGTGATATAAAAGCTGCATATGCCTACAAAGCTCTGAATTCAGGTCCCAGTTGCTGTCA
CAAAGGAGTGAGTGAAACTCCCACCCTACCCCCTTTTTTATATAATAAAAGTGCCTTAGCATGTGTTGCA
GCTGTCACCACTACAGTAAGCTGGTTTACAGATGTTTTCCACTGAGCATCACAATAAAGAGAACCATGT
GCTACGA GCGGCCGC
Mutated
CTCGAGTTTAGAAGAGAGTAGCTCGTCCTCACTGGTCTACACTGGTTGCCGAATTTACTTGTATTCCTAA
CTGTTTTGTATATGCTGCATTGAGACTTACGGCAAGAAGGCATTTTTTTTTTTTAAAGGAAACAAACTCT
CAAATCATGAAGTGATATAAAAGCTGCATATGCCTACAAAGCTCTGAATTCAGGTCCCAGTTGCTGTCA
CAAAGGAGTGAGTGAAACTCCCACCCTACCCCCTTTTTTATATAATAAAAGCATGTGTTGCAGCTGTCAC
CACTACAGTAAGCTGGTTTACAGATGTTTTCCACTGAGCATCACAATAAAGAGAACCATGTGCTACGA
GCGGCCGC
ACTN4 3’UTR-miR-124-3p:NM_004924
GUUGGGGAGACUUGGGGCCAGCGCUUCUGGUCUGGUAAAUAUGUAUGAUGUGUUGUGCUUUU
UUAACCAAGGAGGGGCCAGUGGAUUCCCACAGCACAACCGGUCCCUUCCAUGCCCUGGGAUGCCU
CACCACACCCAGGUCUCUUCCUUUGCUCUGAGGUCCCUUCAAGGCCUCCCCAAUCCAGGCCAAAGC
CCCAUGUGCCUUGUCCAGGAACUGCCUGGGCCAUGCGAGGGGCCAGCAGAGGGCGCCACCACCAC
CUGACGGCUGGGGACCCACCCAGCCCCUCUCCCCUCUCUGCUCCAGACUCACUUGCCAUUGCCAGG
AGAUGGCCCCAACAAGCACCCCGCUUUUGCAGCAGAGGAGCUGAGUUGGCAGACCGGGCCCCCCU
GAACCGCACCCCAUCCCA
Wild-type
CTCGAGGTTGGGGAGACTTGGGGCCAGCGCTTCTGGTCTGGTAAATATGTATGATGTGTTGTGCTTTTT
TAACCAAGGAGGGGCCAGTGGATTCCCACAGCACAACCGGTCCCTTCCATGCCCTGGGATGCCTCACC
ACACCCAGGTCTCTTCCTTTGCTCTGAGGTCCCTTCAAGGCCTCCCCAATCCAGGCCAAAGCCCCATGTG
CCTTGTCCAGGAACTGCCTGGGCCATGCGAGGGGCCAGCAGAGGGCGCCACCACCACCTGACGGCTG
GGGACCCACCCAGCCCCTCTCCCCTCTCTGCTCCAGACTCACTTGCCATTGCCAGGAGATGGCCCCAACA
AGCACCCCGCTTTTGCAGCAGAGGAGCTGAGTTGGCAGACCGGGCCCCCCTGAACCGCACCCCATCCC
AGCGGCCGC
Mutated
CTCGAGGTTGGGGAGACTTGGGGCCAGCGCTTCTGGTCTGGTAAATATGTATGATGTGTTGTGCTTTTT
TAACCAAGGAGGGGCCAGTGGATTCCCACAGCACAACCGGTCCCTTCCATGCCCTGGGATGCCTCACC
ACACCCAGGTCTCTTCCTTTGCTCTGAGGTCCCTTCAAGGCCTCCCCAATCCAGGCCAAAGCCCCATGTC
CAGGAACTGCCTGGGCCATGCGAGGGGCCAGCAGAGGGCGCCACCACCACCTGACGGCTGGGGACCC
ACCCAGCCCCTCTCCCCTCTCTGCTCCAGACTCACTTGCCATTGCCAGGAGATGGCCCCAACAAGCACCC
CGCTTTTGCAGCAGAGGAGCTGAGTTGGCAGACCGGGCCCCCCTGAACCGCACCCCATCCCAGCGGCC
GC

## Slide 4
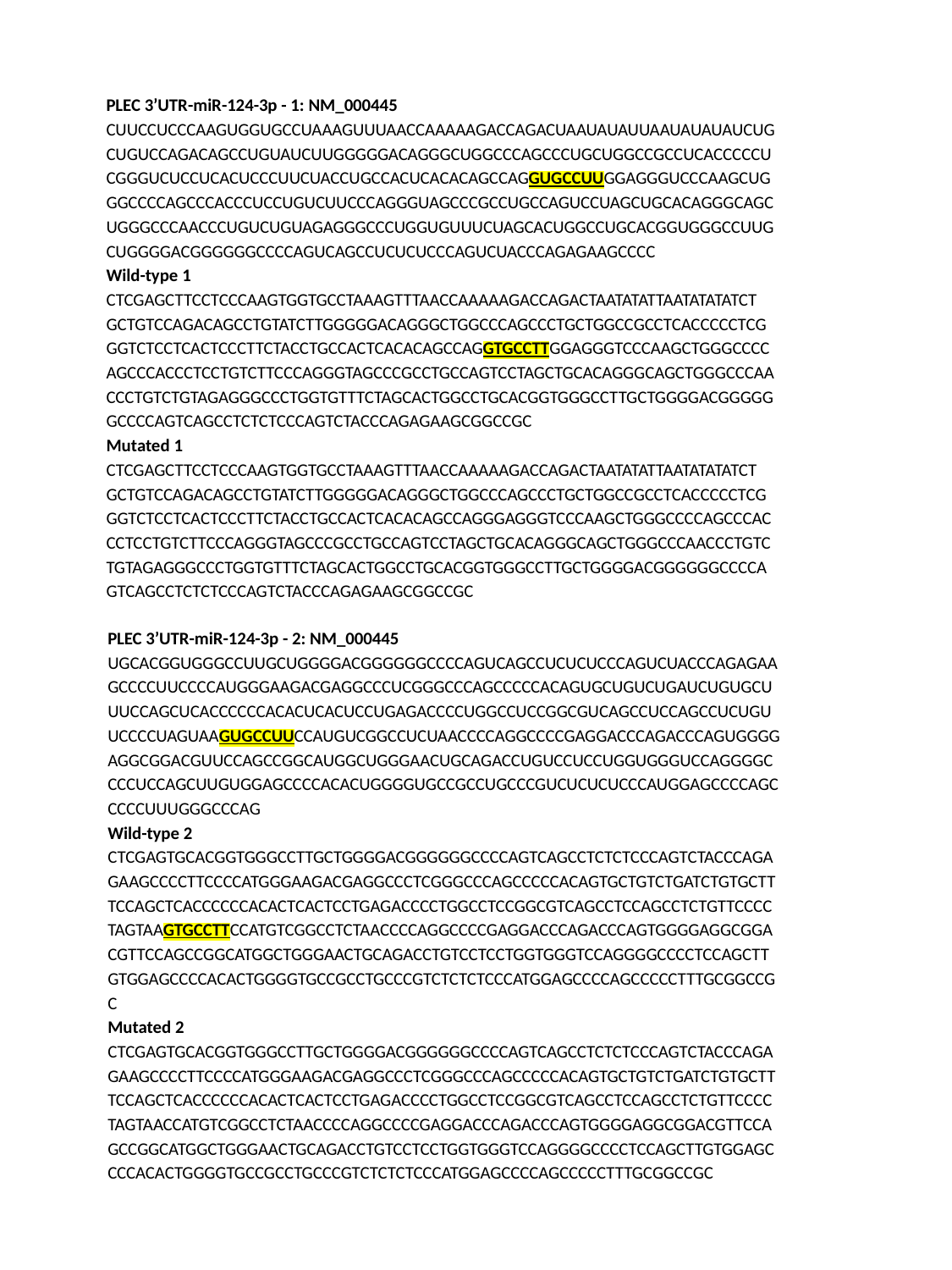

PLEC 3’UTR-miR-124-3p - 1: NM_000445
CUUCCUCCCAAGUGGUGCCUAAAGUUUAACCAAAAAGACCAGACUAAUAUAUUAAUAUAUAUCUG
CUGUCCAGACAGCCUGUAUCUUGGGGGACAGGGCUGGCCCAGCCCUGCUGGCCGCCUCACCCCCU
CGGGUCUCCUCACUCCCUUCUACCUGCCACUCACACAGCCAGGUGCCUUGGAGGGUCCCAAGCUG
GGCCCCAGCCCACCCUCCUGUCUUCCCAGGGUAGCCCGCCUGCCAGUCCUAGCUGCACAGGGCAGC
UGGGCCCAACCCUGUCUGUAGAGGGCCCUGGUGUUUCUAGCACUGGCCUGCACGGUGGGCCUUG
CUGGGGACGGGGGGCCCCAGUCAGCCUCUCUCCCAGUCUACCCAGAGAAGCCCC
Wild-type 1
CTCGAGCTTCCTCCCAAGTGGTGCCTAAAGTTTAACCAAAAAGACCAGACTAATATATTAATATATATCT
GCTGTCCAGACAGCCTGTATCTTGGGGGACAGGGCTGGCCCAGCCCTGCTGGCCGCCTCACCCCCTCG
GGTCTCCTCACTCCCTTCTACCTGCCACTCACACAGCCAGGTGCCTTGGAGGGTCCCAAGCTGGGCCCC
AGCCCACCCTCCTGTCTTCCCAGGGTAGCCCGCCTGCCAGTCCTAGCTGCACAGGGCAGCTGGGCCCAA
CCCTGTCTGTAGAGGGCCCTGGTGTTTCTAGCACTGGCCTGCACGGTGGGCCTTGCTGGGGACGGGGG
GCCCCAGTCAGCCTCTCTCCCAGTCTACCCAGAGAAGCGGCCGC
Mutated 1
CTCGAGCTTCCTCCCAAGTGGTGCCTAAAGTTTAACCAAAAAGACCAGACTAATATATTAATATATATCT
GCTGTCCAGACAGCCTGTATCTTGGGGGACAGGGCTGGCCCAGCCCTGCTGGCCGCCTCACCCCCTCG
GGTCTCCTCACTCCCTTCTACCTGCCACTCACACAGCCAGGGAGGGTCCCAAGCTGGGCCCCAGCCCAC
CCTCCTGTCTTCCCAGGGTAGCCCGCCTGCCAGTCCTAGCTGCACAGGGCAGCTGGGCCCAACCCTGTC
TGTAGAGGGCCCTGGTGTTTCTAGCACTGGCCTGCACGGTGGGCCTTGCTGGGGACGGGGGGCCCCA
GTCAGCCTCTCTCCCAGTCTACCCAGAGAAGCGGCCGC
PLEC 3’UTR-miR-124-3p - 2: NM_000445
UGCACGGUGGGCCUUGCUGGGGACGGGGGGCCCCAGUCAGCCUCUCUCCCAGUCUACCCAGAGAA
GCCCCUUCCCCAUGGGAAGACGAGGCCCUCGGGCCCAGCCCCCACAGUGCUGUCUGAUCUGUGCU
UUCCAGCUCACCCCCCACACUCACUCCUGAGACCCCUGGCCUCCGGCGUCAGCCUCCAGCCUCUGU
UCCCCUAGUAAGUGCCUUCCAUGUCGGCCUCUAACCCCAGGCCCCGAGGACCCAGACCCAGUGGGG
AGGCGGACGUUCCAGCCGGCAUGGCUGGGAACUGCAGACCUGUCCUCCUGGUGGGUCCAGGGGC
CCCUCCAGCUUGUGGAGCCCCACACUGGGGUGCCGCCUGCCCGUCUCUCUCCCAUGGAGCCCCAGC
CCCCUUUGGGCCCAG
Wild-type 2
CTCGAGTGCACGGTGGGCCTTGCTGGGGACGGGGGGCCCCAGTCAGCCTCTCTCCCAGTCTACCCAGA
GAAGCCCCTTCCCCATGGGAAGACGAGGCCCTCGGGCCCAGCCCCCACAGTGCTGTCTGATCTGTGCTT
TCCAGCTCACCCCCCACACTCACTCCTGAGACCCCTGGCCTCCGGCGTCAGCCTCCAGCCTCTGTTCCCC
TAGTAAGTGCCTTCCATGTCGGCCTCTAACCCCAGGCCCCGAGGACCCAGACCCAGTGGGGAGGCGGA
CGTTCCAGCCGGCATGGCTGGGAACTGCAGACCTGTCCTCCTGGTGGGTCCAGGGGCCCCTCCAGCTT
GTGGAGCCCCACACTGGGGTGCCGCCTGCCCGTCTCTCTCCCATGGAGCCCCAGCCCCCTTTGCGGCCG
C
Mutated 2
CTCGAGTGCACGGTGGGCCTTGCTGGGGACGGGGGGCCCCAGTCAGCCTCTCTCCCAGTCTACCCAGA
GAAGCCCCTTCCCCATGGGAAGACGAGGCCCTCGGGCCCAGCCCCCACAGTGCTGTCTGATCTGTGCTT
TCCAGCTCACCCCCCACACTCACTCCTGAGACCCCTGGCCTCCGGCGTCAGCCTCCAGCCTCTGTTCCCC
TAGTAACCATGTCGGCCTCTAACCCCAGGCCCCGAGGACCCAGACCCAGTGGGGAGGCGGACGTTCCA
GCCGGCATGGCTGGGAACTGCAGACCTGTCCTCCTGGTGGGTCCAGGGGCCCCTCCAGCTTGTGGAGC
CCCACACTGGGGTGCCGCCTGCCCGTCTCTCTCCCATGGAGCCCCAGCCCCCTTTGCGGCCGC

## Slide 5
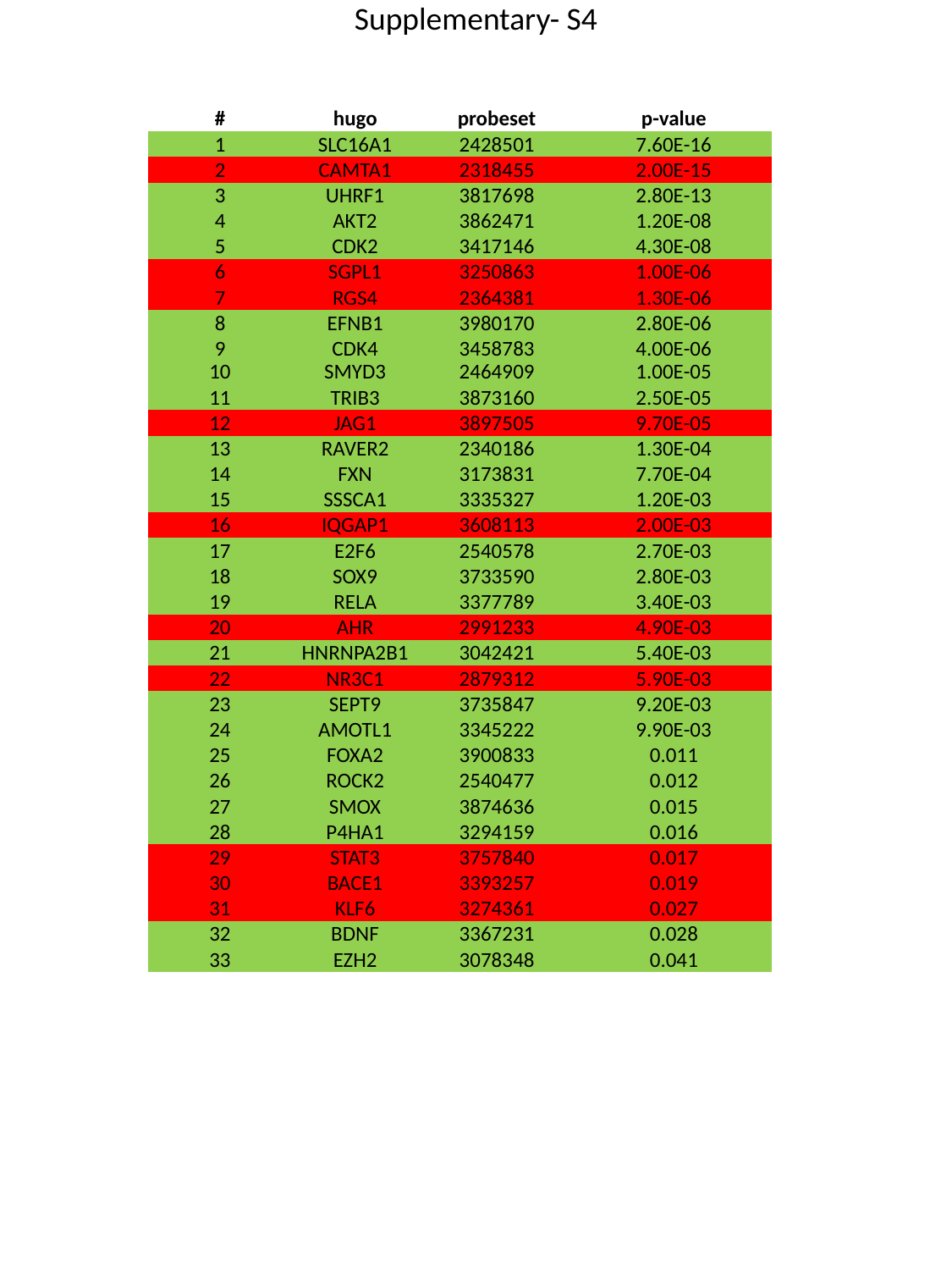

# Supplementary- S4
| # | hugo | probeset | p-value |
| --- | --- | --- | --- |
| 1 | SLC16A1 | 2428501 | 7.60E-16 |
| 2 | CAMTA1 | 2318455 | 2.00E-15 |
| 3 | UHRF1 | 3817698 | 2.80E-13 |
| 4 | AKT2 | 3862471 | 1.20E-08 |
| 5 | CDK2 | 3417146 | 4.30E-08 |
| 6 | SGPL1 | 3250863 | 1.00E-06 |
| 7 | RGS4 | 2364381 | 1.30E-06 |
| 8 | EFNB1 | 3980170 | 2.80E-06 |
| 9 | CDK4 | 3458783 | 4.00E-06 |
| 10 | SMYD3 | 2464909 | 1.00E-05 |
| 11 | TRIB3 | 3873160 | 2.50E-05 |
| 12 | JAG1 | 3897505 | 9.70E-05 |
| 13 | RAVER2 | 2340186 | 1.30E-04 |
| 14 | FXN | 3173831 | 7.70E-04 |
| 15 | SSSCA1 | 3335327 | 1.20E-03 |
| 16 | IQGAP1 | 3608113 | 2.00E-03 |
| 17 | E2F6 | 2540578 | 2.70E-03 |
| 18 | SOX9 | 3733590 | 2.80E-03 |
| 19 | RELA | 3377789 | 3.40E-03 |
| 20 | AHR | 2991233 | 4.90E-03 |
| 21 | HNRNPA2B1 | 3042421 | 5.40E-03 |
| 22 | NR3C1 | 2879312 | 5.90E-03 |
| 23 | SEPT9 | 3735847 | 9.20E-03 |
| 24 | AMOTL1 | 3345222 | 9.90E-03 |
| 25 | FOXA2 | 3900833 | 0.011 |
| 26 | ROCK2 | 2540477 | 0.012 |
| 27 | SMOX | 3874636 | 0.015 |
| 28 | P4HA1 | 3294159 | 0.016 |
| 29 | STAT3 | 3757840 | 0.017 |
| 30 | BACE1 | 3393257 | 0.019 |
| 31 | KLF6 | 3274361 | 0.027 |
| 32 | BDNF | 3367231 | 0.028 |
| 33 | EZH2 | 3078348 | 0.041 |

## Slide 6
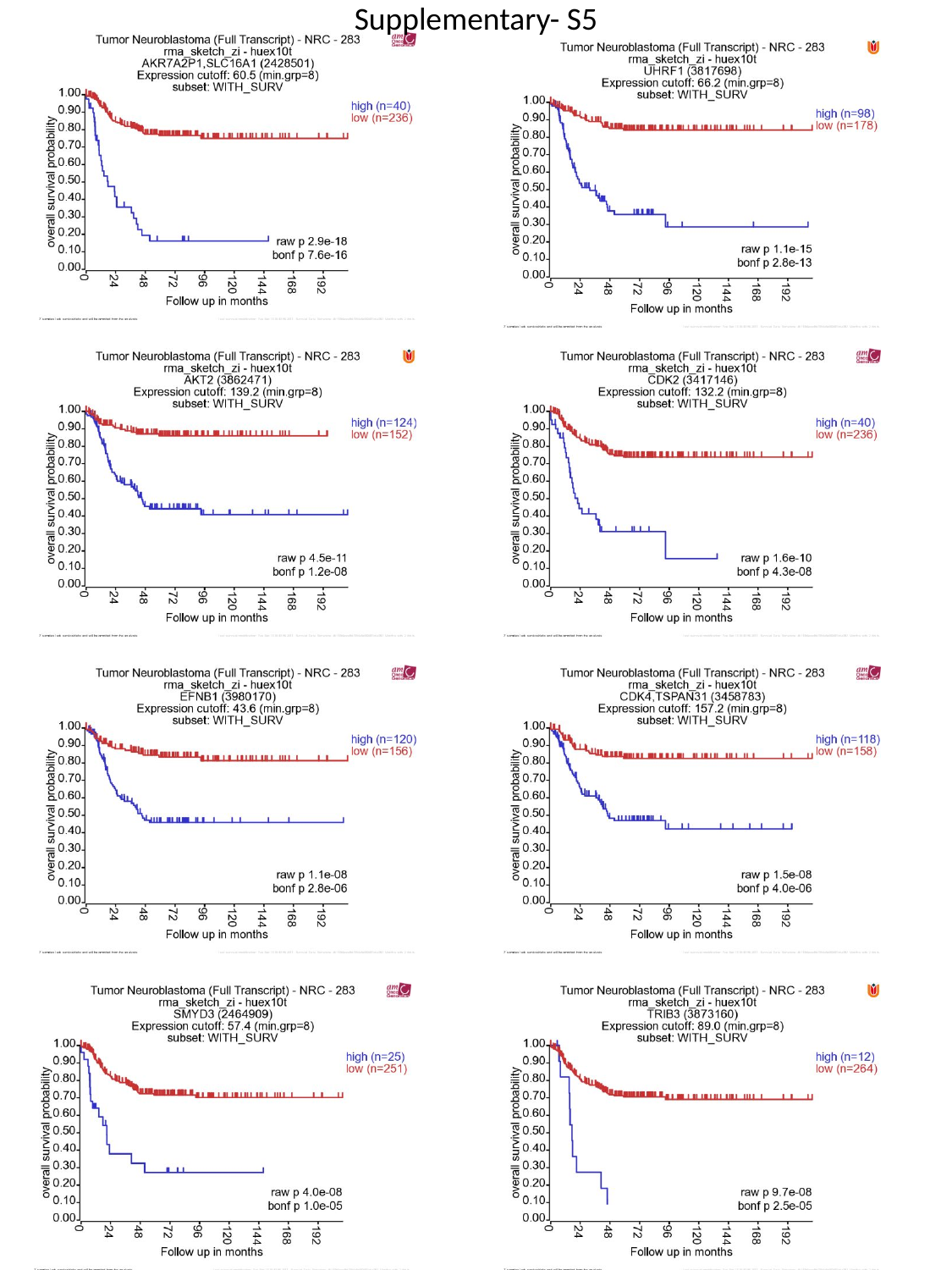

# Supplementary- S5

## Slide 7
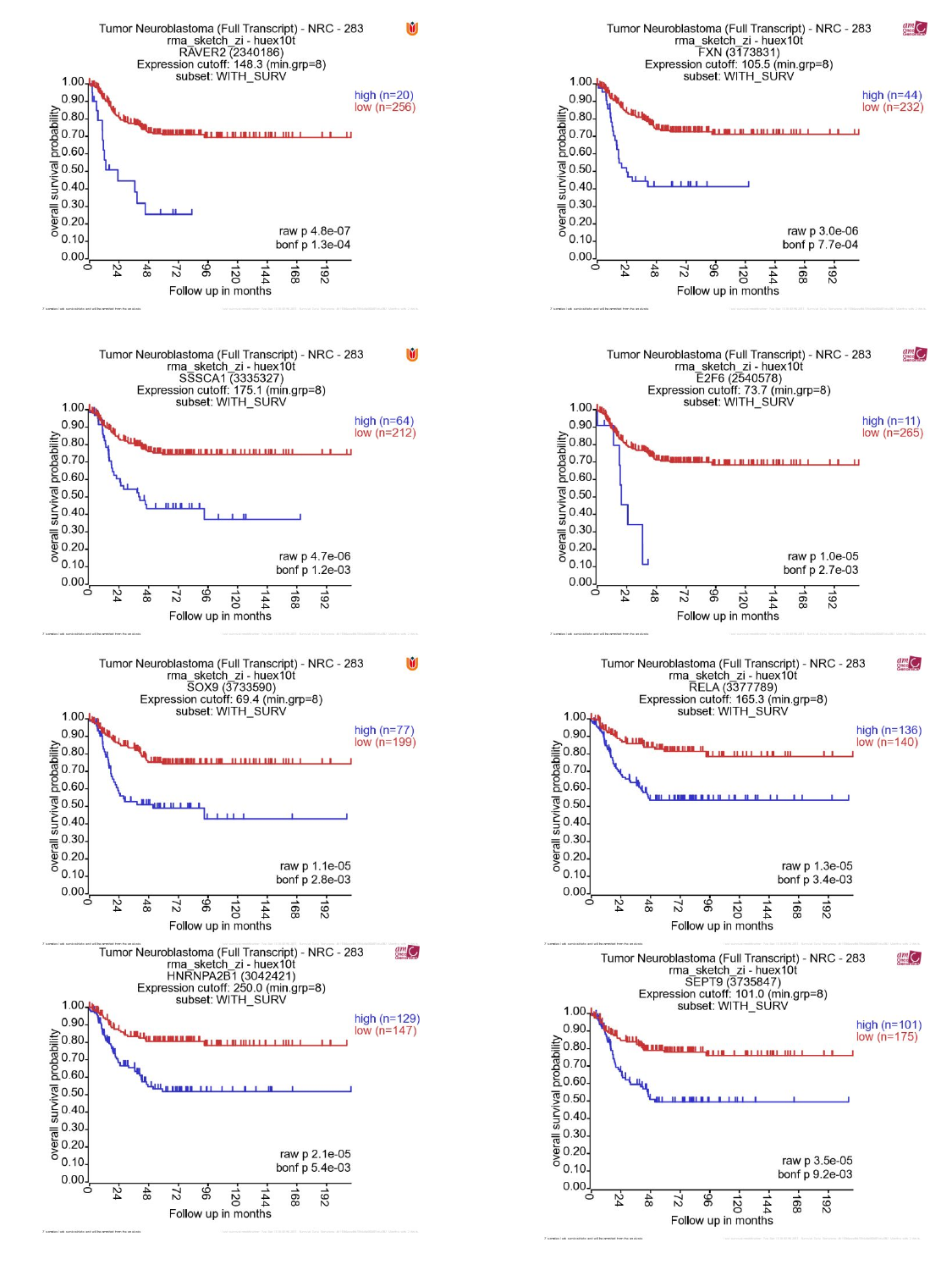

## Slide 8
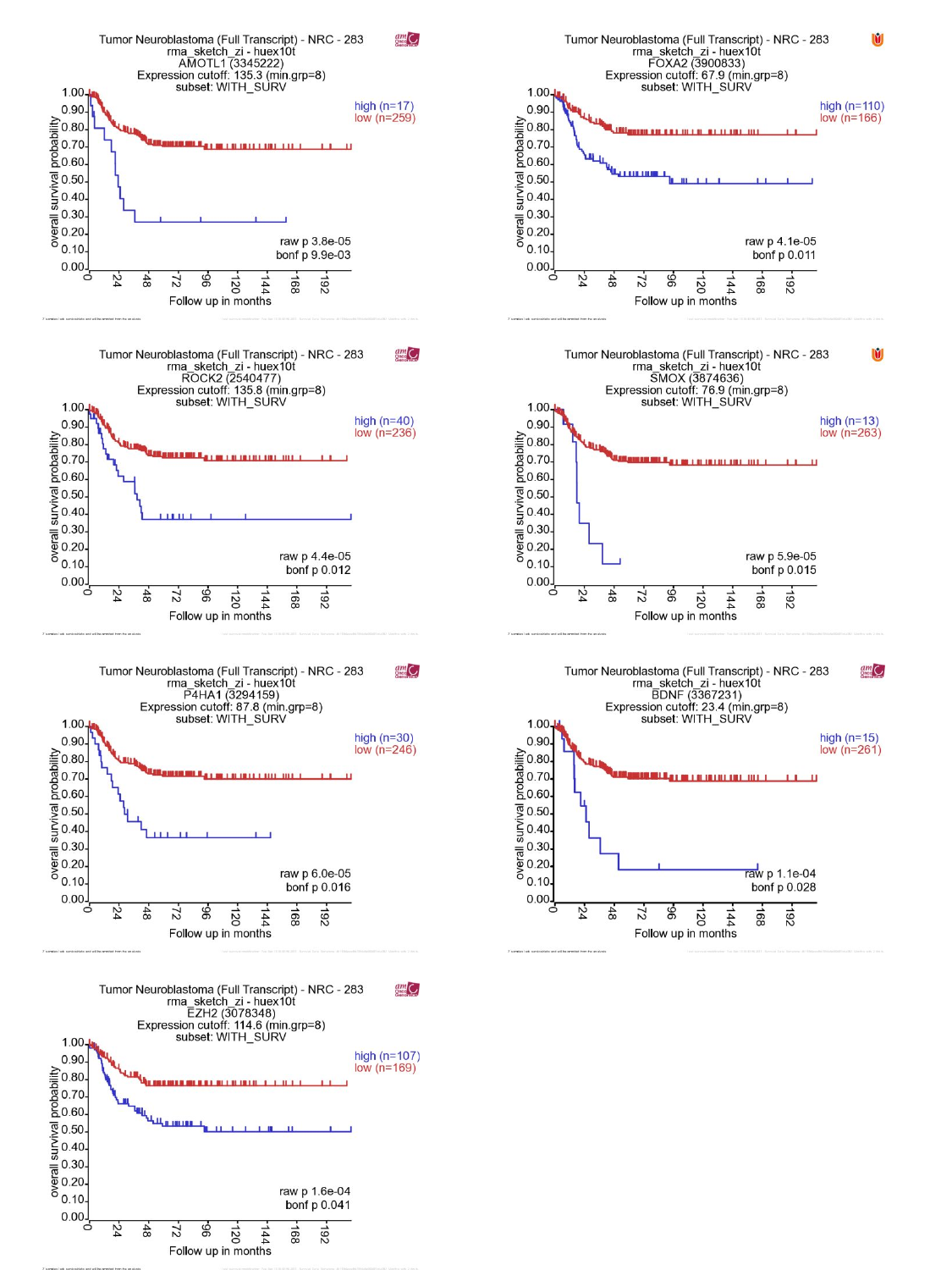

## Slide 9
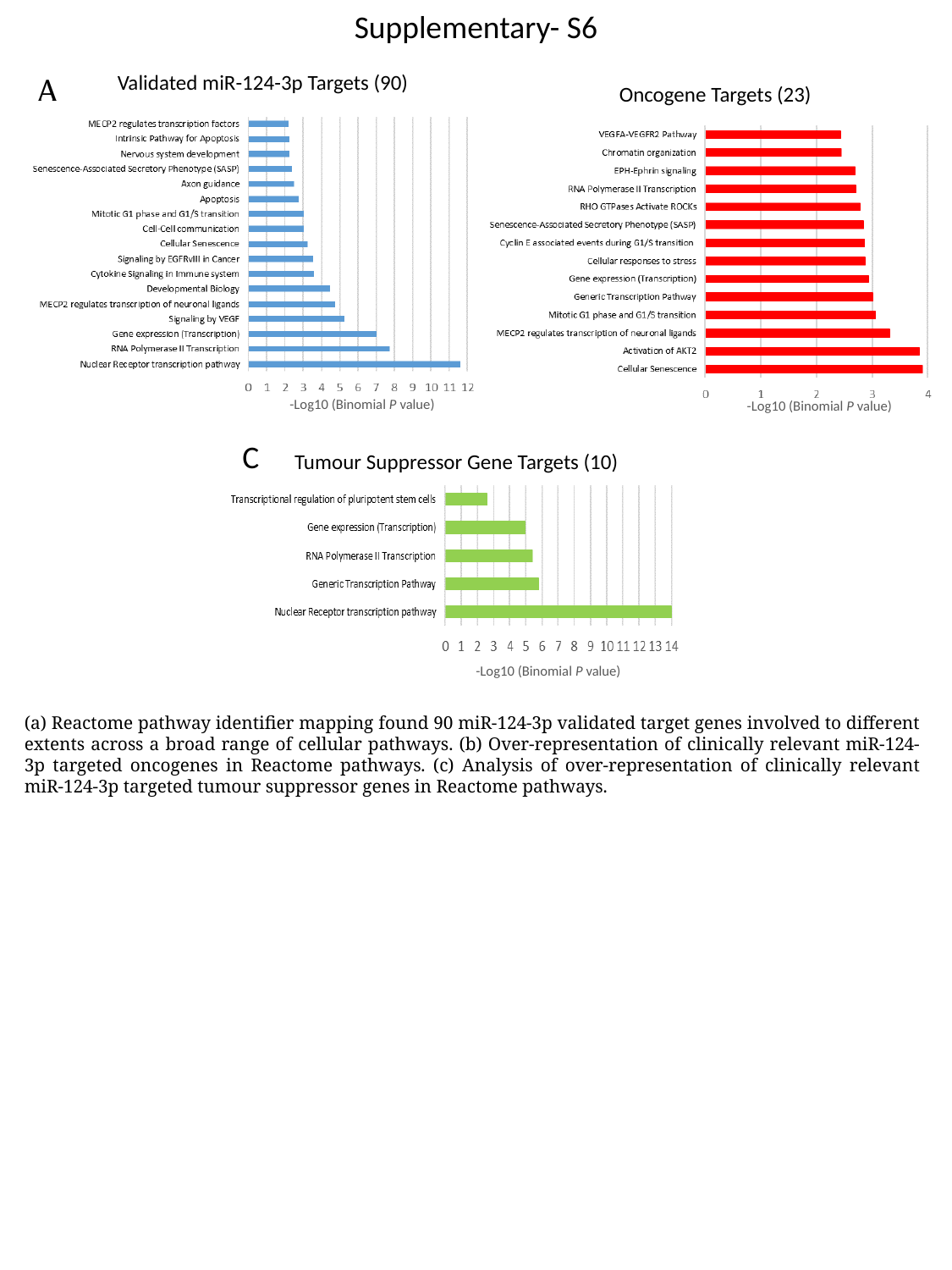

# Supplementary- S6
Validated miR-124-3p Targets (90)
A
Oncogene Targets (23)
-Log10 (Binomial P value)
-Log10 (Binomial P value)
C
Tumour Suppressor Gene Targets (10)
-Log10 (Binomial P value)
(a) Reactome pathway identifier mapping found 90 miR-124-3p validated target genes involved to different extents across a broad range of cellular pathways. (b) Over-representation of clinically relevant miR-124-3p targeted oncogenes in Reactome pathways. (c) Analysis of over-representation of clinically relevant miR-124-3p targeted tumour suppressor genes in Reactome pathways.

## Slide 10
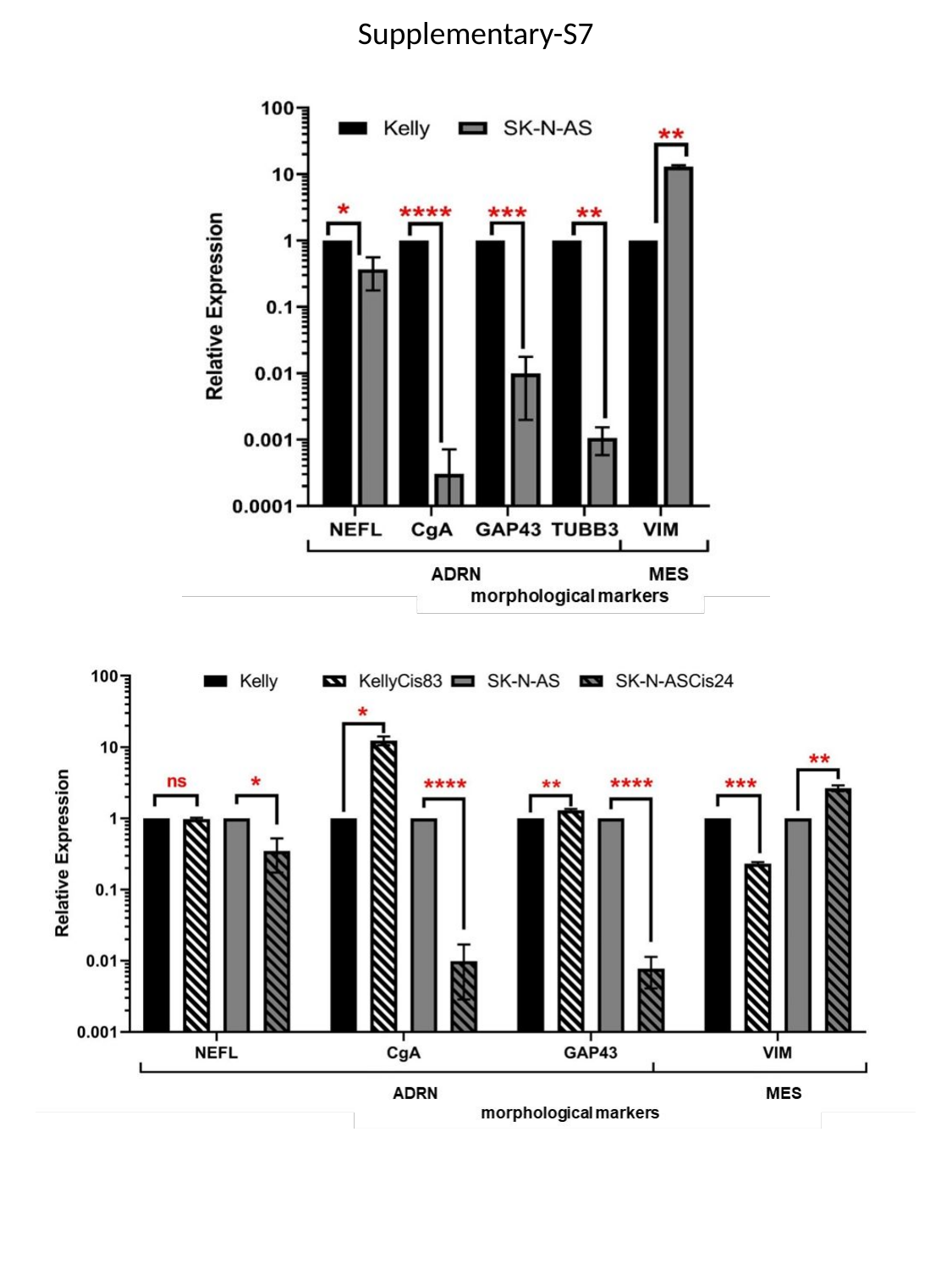

Supplementary-S7
